# Selective perturbation of mirror and non-mirror neurons in an in silico model of the action observation network

**DOI:** 10.1016/j.isci.2026.116776

**Published:** 2026-07-17

**Authors:** Luca Guglielmi, Davide Albertini, Alessandro Vezzani, Raffaella Burioni, Luca Bonini

**Affiliations:** 1 Department of Mathematical, Physical and Computer Sciences, University of Parma, Parma, Italy; 2 INFN - Parma Unit, Parma, Italy; 3 Department of Medicine and Surgery, University of Parma, Parma, Italy; 4 IMEM-CNR, Parma, Italy

**Keywords:** recurrent neural network, mirror neuron, action observation, motor system, computational modeling

## Abstract

The action observation network (AON) is a distributed system of brain areas containing mirror neurons (MNs), active during both action execution and observation, as well as purely motor, non-mirror neurons. Here, we present a biologically inspired recurrent neural network (RNN) model trained to reproduce single-neuron activity from three key macaque AON areas—anterior intraparietal area (AIP), areas F5 and F6. The model accurately captured experimental firing patterns and enabled the reconstruction of candidate functional connectivity. Cell-type-specific in silico silencing revealed that inhibitory interneurons exert a strong influence on network function, with those in F5 and F6 contributing to agent identity discrimination. Silencing excitatory non-mirror neurons in F5 and F6 produced larger reductions in self-action decoding performance, whereas silencing excitatory MNs in AIP preferentially impacted the decoding of others’ actions. These findings provide a computational framework for linking cell-type-specific perturbations to population-level action representations in the AON.

## Highlights

• Constrained RNNs model macaque AON activity during execution and observation
• Silencing inhibitory neurons disrupts network dynamics and agent identity decoding
• Excitatory non-mirror neurons in F5 and F6 support self-action decoding
• Excitatory mirror neurons in AIP preferentially support other-action decoding

## Graphical abstract

**Figure:**
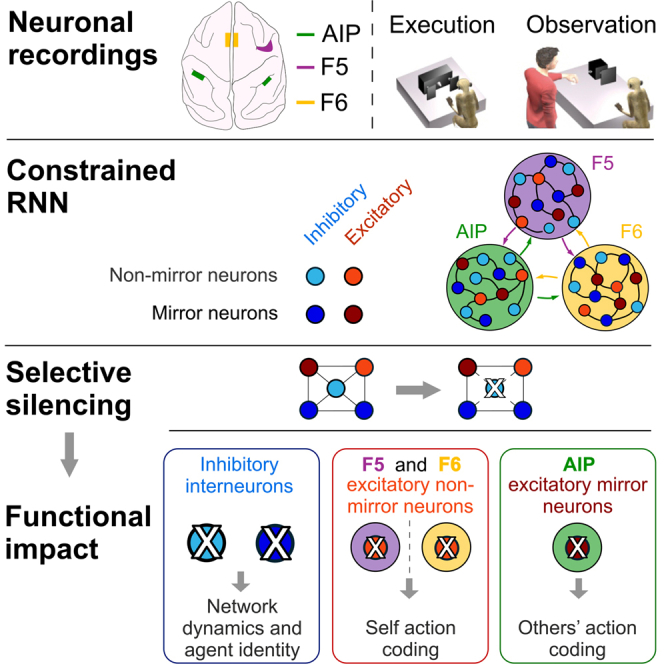

## Teaser

Biocomputational method; Neuroscience

## Introduction

Mirror neurons (MNs) are a class of cells discovered in the macaque premotor cortex that discharge during action execution and during the observation of actions performed by others.^1^ Studies on monkeys and humans have revealed the presence of cells with mirror properties in a wide network of brain areas,^2^^,^^3^^,^^4^ known as the action observation network (AON). Additionally, studies on other animal models have demonstrated that, beyond the order of primates, this mechanism is present in birds^5^ and rodents,^6^^,^^7^ suggesting that it is phylogenetically more ancient and widespread than originally thought.

After three decades of research and the vast influence of MN literature on social and cognitive neuroscience,^8^ there is no causal neurophysiological evidence on the role of different cell classes on the functioning of the AON. This is due to at least two main reasons. First, the original proposal that MNs implement “action understanding” through direct visuomotor mapping of the observed action onto one’s own motor representations^9^ did not provide an independent definition of “understanding,” making it difficult to formulate testable predictions from the cellular to the behavioral level. Indeed, studies that have tried to empirically test action understanding ended up investigating a variety of processes,^10^ such as action recognition,^11^ discrimination,^12^ or prediction.^13^ Second, MNs in primates constitute a highly heterogeneous population: some are pyramidal corticospinal neurons,^14^ others may be inhibitory interneurons,^15^ and all these cells are intermingled with other motor and visuomotor non-mirror neurons in a vast bilateral parieto-frontal system, possibly involving subcortical structures as well.^3^ This makes it extremely difficult to practically operate meaningful and relevant perturbation experiments because the available causal techniques are particularly effective for targeting neuronal populations of a specific type and/or anatomically localized, such as place cells^16^ or face-selective cells.^17^

Recurrent neural network (RNN) models have proven effective in capturing regional and cell-type specificities in brain activity.^18^^,^^19^^,^^20^ Typically, such models are trained in a supervised and goal-directed manner to execute specific tasks, and comparisons between *in vivo* and in silico neural population dynamics are then exploited to infer general computational principles.^21^^,^^22^^,^^23^^,^^24^ Related recurrent modeling approaches have also been used to study mirror-system-like sensorimotor transformations.^25^

Given the absence of an overt motor task during action observation and the impossibility of directly testing the behavioral function of MNs in monkeys, we built a biologically inspired, continuous-variable rate RNN model trained to replicate experimentally recorded single-unit firing rates across the AON.^15^^,^^26^^,^^27^^,^^28^^,^^29^ This approach allowed us to reconstruct the complex neuronal dynamics of the AON with high fidelity, providing a flexible computational framework that captures activity patterns across both execution and observation conditions and enables selective in silico perturbations of identified neuronal classes—mirror versus non-mirror and excitatory versus inhibitory—currently unachievable *in vivo*. Specifically, we aimed to determine how the architecture of the AON, when trained to reproduce empirical neuronal dynamics, organizes its intra- and inter-areal synaptic connectivity, and how the silencing of distinct neuronal types affects the population-level representation of self- and other-related actions.

The results highlight a central role of inhibitory neurons in shaping task-specific neural dynamics within the AON. Regional differences emerged, with motor areas (i.e., F5 and F6) exhibiting an overall stronger impact on network functioning. Selective in silico cell-type perturbations revealed that silencing excitatory non-mirror neurons in F5 and F6 produced larger reductions in self-action decoding performance, whereas silencing excitatory MNs in AIP had a stronger effect on the decoding of others’ actions. Notably, agent-decoding analyses revealed a prominent role of inhibitory neurons in F5 and F6, whose silencing markedly impaired the discrimination between executed and observed actions.

These findings are consistent with a functionally differentiated organization across AON regions and neuronal classes, paving the way to future in silico simulations of the extended AON functioning.

## Results

### Trained RNNs replicate experimental single-neuron and population-level dynamics of the AON with high fidelity

**Figure 1:**
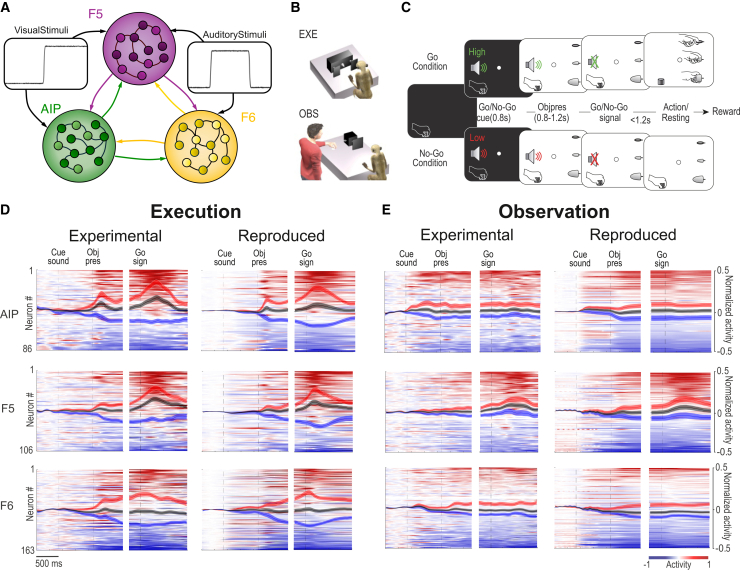
RNN model trained to replicate experimental data (A) Schematic representation of the network comprising the three interconnected regions AIP, F5, and F6, along with the external inputs they receive. Visual stimuli, modeling exposure to one of the three objects, project to AIP and F5. Auditory stimuli, representing the Go or No-Go cues, project to F6 and F5. (B) Schematic representation of the execution (top) and observation (bottom) contexts. (C) Temporal sequence of the experimental task: following the Go or No-Go cue, the light is turned on and one of three possible objects is presented to the monkey. Upon cessation of the auditory cue, the monkey is expected to either grasp the object in response to Go or maintain fixation in response to No-Go. Following completion of the task, the monkey receives a liquid reward. (D) Comparison of recorded (left) and reproduced (right) neural dynamics during the Go Execution task, organized by brain area (AIP, top; F5, middle; F6, bottom). Reproduced data refer to a representative RNN (*R* = 0.948) selected from 20 independently trained networks. Each row in the heatmaps shows the average activity of a single neuron across the three object conditions. Neurons are sorted from top to bottom by the magnitude of their activity relative to baseline, in the interval between 300 ms before and 900 ms after the Go signal, separately for recorded and reproduced data. Red indicates increased activity, and blue indicates suppression. Solid lines represent the mean activity of neurons with increased (red) or suppressed (blue) responses and the overall population average (black). (E) Same as (D), but for the Go observation task.

To build an in silico model of the AON (Figure 1A), we employed a continuous-variable rate RNN (see STAR Methods). This model was designed to replicate the single-neuron firing patterns observed in previous neurophysiological recordings,^15^^,^^29^ which were obtained during tasks in which monkeys both executed and observed (Figure 1B) a Go/No-Go reaching-grasping task (Figure 1C). The model consisted of 355 neurons, corresponding one-to-one with the neurons of the experimental dataset (86 in AIP, 106 in F5, and 163 in F6).

The dynamics of the system are governed by a set of differential equations that simulate the temporal evolution of the synaptic current *x*_*i*_ and firing rate *r*_*i*_ of each neuron^30^^,^^31^^,^^32^:

(Equation 1) $$\tau_{i}^{d}\;\frac{dx_{i}}{dt}=-\;x_{i}+\overset{N}{\underset{j=1}{\sum }}w_{ij}\;\cdot \;\phi \left(x_{j}\right)+I_{\mathrm{ext}}$$

(Equation 2) $$r_{i}=\phi \left(x_{i}\right),$$

where $\tau_{i}^{d}$ is the synaptic decay time constant for unit *i*, (*i* = 1, 2, …, *N*, *N* being the total number of neurons in the network). The firing rate was determined by applying a nonlinear activation function *ϕ*(*x*)—specifically, a standard sigmoid function—to the synaptic current (Equation 4). External currents *I*_*ext*_ included task-specific stimuli to simulate the experimental conditions. *W*_*ij*_ is the element of the synaptic connectivity matrix *W* that describes the interaction from unit *j* to unit *i*. This matrix provides a detailed representation of the interactions within the neural network, with positive and negative values indicating excitatory and inhibitory connections, respectively.

We trained the RNN to minimize the difference between generated and recorded neural activity for all 12 task conditions (Figure 1D). Owing to the high dimensionality of the parameter space, training does not yield a unique solution but rather a family of functionally similar solutions of interneuron connectivity capable of reproducing the observed dynamics. To evaluate the consistency and generality of our findings, we independently trained multiple networks from different random initializations and verified that all converged to qualitatively similar solutions. These networks consistently achieved a high degree of accuracy in reproducing experimental firing patterns (*mean* ± *SD*: *RMSE* = 0.0195 ± 0.0012; global population-level Pearson correlation coefficient *R* = 0.943 ± 0.007; Table S1).

The global structure of the simulated population dynamics was further examined through principal-component analysis (PCA), which confirmed that the recurrent network captured the dominant temporal features of the experimental activity (see Figure S1). The first two principal components explained (mean ± SD) 60.4 ± 4.4% of the variance in the experimental activity and 77.5 ± 4.7% in the synthetic activity, indicating that PC1–PC2 projection captures a substantial fraction of the population dynamics in both cases.

### Learned network topology reveals structured recurrent organization and local inhibitory clustering

To investigate the structure underlying our RNNs’ functioning, we initialized the connectivity matrix *W* of each RNN by imposing minimal constraints, thereby ensuring sufficient flexibility for the emergence of structural properties during training. Specifically, each unit in the network was classified as inhibitory or excitatory on the basis of the spike shape and firing characteristics exhibited by its biological counterpart in the experimental dataset^15^ (see STAR Methods). Additionally, inhibitory connections were restricted to neurons within the same anatomical region, in accordance with anatomical evidence.^33^ These constraints were introduced to maximize the biological plausibility of the model.^28^

**Figure 2:**
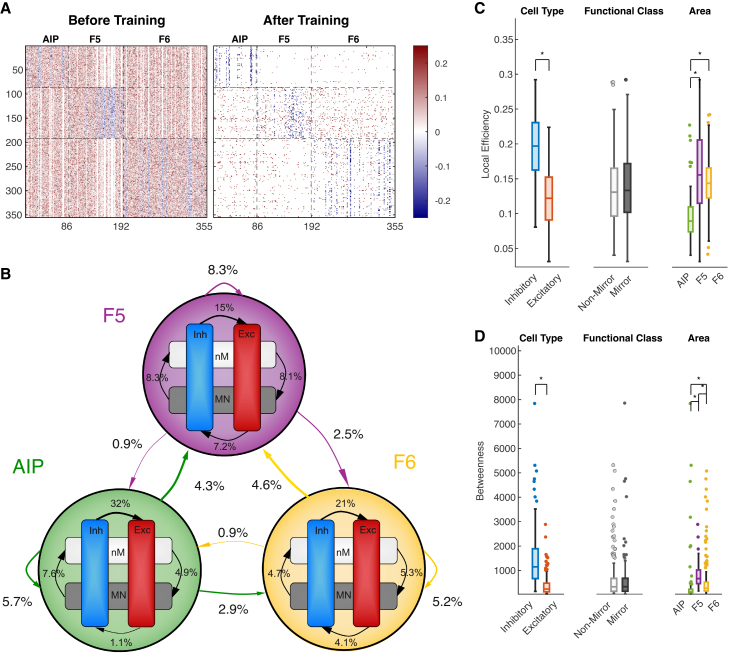
Connectivity matrix evolution and network metrics (A) Directed connectivity matrix before (left) and after (right) training for a representative RNN. Each matrix element *w*_*ij*_ represents the synaptic connection from presynaptic neuron *j* (column) to postsynaptic neuron *i* (row). Inhibitory weights are shown in blue, excitatory weights in red, and null connections in white. Neurons are ordered by anatomical region—AIP (1–86), F5 (87–192), and F6 (193–355)—as indicated by the dashed black lines. (B) Inter- and intra-area connectivity among AIP, F5, and F6. Percentages indicate the fraction of existing (nonzero) connections relative to all possible ones for each area pair, excluding connections that were constrained to be absent by design. Values represent mean connection probabilities across the 20 independently trained networks (see Tables S2 and S3 for detailed statistics). Intra-area connectivity is further detailed separately for cell type—excitatory (Exc, red) and inhibitory (Inh, blue)—and for functional class—mirror (MN, dark shades) and non-mirror (nM, light shades). (C) Distribution of local efficiency across different neuron classifications. Boxplots show comparisons based on cell type, excitatory vs. inhibitory (left); functional class, mirror vs. non-mirror (center); and anatomical area, AIP, F5, and F6 (right). In each boxplot, the central thick line represents the median, while the box spans the interquartile range (IQR), defined as the range between the first (25th percentile) and third quartile (75th percentile). The whiskers extend to the most extreme data points within 1.5 times the IQR from the first and third quartiles. Data points beyond this range are shown as individual dots and represent outliers. Asterisks indicate significant differences (*p* < 0.05, Mann-Whitney U test) between the corresponding groups. Exact *p* values are reported in the main text. (D) Same as (C), but for the betweenness centrality.

The connectivity matrix was initially set up as a fully connected one. After training, *W* evolved into a sparse structure (Figure 2A) in all independently trained networks.^34^ The trained networks exhibited remarkable consistency in their global structural properties. In particular, the overall connectivity, defined as the fraction of active synaptic connections relative to the total number of possible connections, was highly consistent across networks (Figure 2B). Possible connections were defined as all nonzero entries in the weight matrix *W* that were not fixed to zero during model construction (see STAR Methods).

Next, we tested whether the inferred connectivity matrices resulting from the training procedure exhibited hallmark features consistent with their anatomical counterparts. In agreement with anatomical evidence from macaque tracing studies,^35^ the connectivity within each area was stronger than connectivity between areas. To quantify the possible directional asymmetry of inter-areal connectivity, we computed the asymmetry index (also referred to as the balance ratio^36^^,^^37^), defined for each area *k* as:

(Equation 3) $$AI_{k}=\frac{Out_{k}-In_{k}}{Out_{k}+In_{k}},$$

where *Out*_*k*_ and *In*_*k*_ indicate the number of total outgoing and incoming connections of area *k*, respectively. The results revealed that AIP showed a positive balance ratio (*AI*_*AIP*_ = 0.60 ± 0.03), i.e., predominance of efferent over afferent connections, whereas F5 had a negative asymmetry index (*AI*_*F*5_ = −0.48 ± 0.04), and F6 was approximately balanced (*AI*_*F*6_ = 0.09 ± 0.04). Although cortico-cortical connexions are generally bidirectional, this finding is in line with the previously proposed role of AIP as a functional hub for routing visual information to other nodes of the AON.^38^^,^^39^ At the cellular level, the asymmetry index was positive for inhibitory neurons (*AI*_Inh_ = 0.18 ± 0.03), indicating that they formed more outgoing connections toward excitatory neurons than they received from them. By definition, the asymmetry index for excitatory neurons (*AI*_Exc_) is the negative counterpart of that for inhibitory neurons, such that *AI*_Exc_ = −*AI*_Inh_.

Notably, training the network separately on execution and observation data largely preserved the overall synaptic connectivity density observed under joint training (Figures S2E and S2F). This stability across the two contexts supports the robustness of the learned functional architecture across training regimes, indicating that the model captures organizational features consistent with known properties of the AON that remain consistent across behavioral contexts.

We further analyzed the architecture emerging from RNN training by computing, for each neuron, two connectivity metrics: local efficiency, which quantifies the degree of connectivity among a neuron’s immediate neighbors, and betweenness centrality, which measures how often a neuron lies on the shortest paths between other neuron pairs (see STAR Methods). Differences between groups were evaluated using two-sided Mann-Whitney U tests. Each neuron was treated as an independent observation and for each neuron, the metric of interest was averaged across the 20 trained networks before comparison.

Our findings revealed that inhibitory neurons showed higher local efficiency (*p* < 10^−20^) (Figure 2C), a greater number of both incoming (*p* < 10^−22^) and outgoing connections (*p* < 10^−9^) (Figures S2A and S2B), and higher betweenness (*p* < 10^−22^) (Figure 2D) values compared with excitatory pyramidal neurons, consistent with dense local inhibitory clustering typical of cortical regions.^40^^,^^41^ Because the recurrent architecture included biological constraints, most notably the restriction of inhibitory projections within local cortical areas, we examined whether the observed topological differences were already present before training or emerged during optimization. Before training, inhibitory neurons already showed higher local efficiency, consistent with the imposed local-connectivity constraint, whereas their betweenness centrality was minimal (Figure S2C). However, analysis of training-induced changes revealed a training-dependent reorganization of the topology: inhibitory neurons showed a weaker reduction in local efficiency and a stronger increase in betweenness centrality than excitatory neurons (Figure S2D). Thus, local clustering was partly related to the initial architectural constraint, whereas the increased centrality of inhibitory neurons emerged primarily through training. No significant differences were observed between mirror and non-mirror neurons (local efficiency: *p* = 0.58; in-degree: *p* = 0.71; out-degree: *p* = 0.54; betweenness: *p* = 0.95) (Figure 2D). When comparing anatomical areas, both local efficiency and betweenness centrality differed significantly across regions: area F5 exhibited the highest values, followed by F6, and AIP showing the lowest values (local efficiency: AIP–F5, *p* < 10^−11^; AIP–F6, *p* < 10^−19^; F5–F6, *p* = 0.095; betweenness centrality: AIP–F5, *p* < 10^−20^; AIP–F6, *p* < 10^−13^; F5–F6, *p* < 10^−9^).

We also evaluated the tendency of neurons to preferentially connect with other cells of the same type (e.g., mirror-to-mirror) by computing Newman’s assortativity coefficient (Equation 12), a standard metric for quantifying homophily in network connectivity.^36^^,^^42^ For inhibitory and excitatory neurons, we found a negative assortativity (*r* = −0.17 ± 0.02), revealing a preference for cross-type connections. In contrast, the assortativity between mirror and non-mirror neurons was near zero (*r* = 0.01 ± 0.01), indicating a similar tendency to establish connections within and between the two classes.

Because execution and observation were implemented through distinct input matrices acting on a shared recurrent architecture, we analyzed the corresponding input weights. Execution- and observation-related input weights differed across all neuronal populations except for auditory input to non-mirror neurons in F6. These differences were more pronounced for visual inputs to AIP and F5 (see Figure S3).

### Cell-type-specific perturbations reveal distinct contributions to task-related network dynamics

We leveraged the trained RNNs to simulate experiments so far impractical to perform *in vivo*, such as the selective inactivation of specific units of the network by setting their firing rates to zero throughout the activity generation process. This reproduces the GABAergic tone enhancement achieved by muscimol experiments^43^ but with single-cell resolution, which is empirically hardly achievable in non-human primates to date. Then, to quantify the impact of silencing on the network functioning, we trained a bidirectional long short-term memory classifier (see STAR Methods) to distinguish firing rate patterns corresponding to specific task conditions and measured the classification accuracy as a function of the number and class of inactivated units. Statistical differences between silencing curves corresponding to different neuronal groups were then assessed within each decoder through permutation-based comparisons at each silencing level.

**Figure 3:**
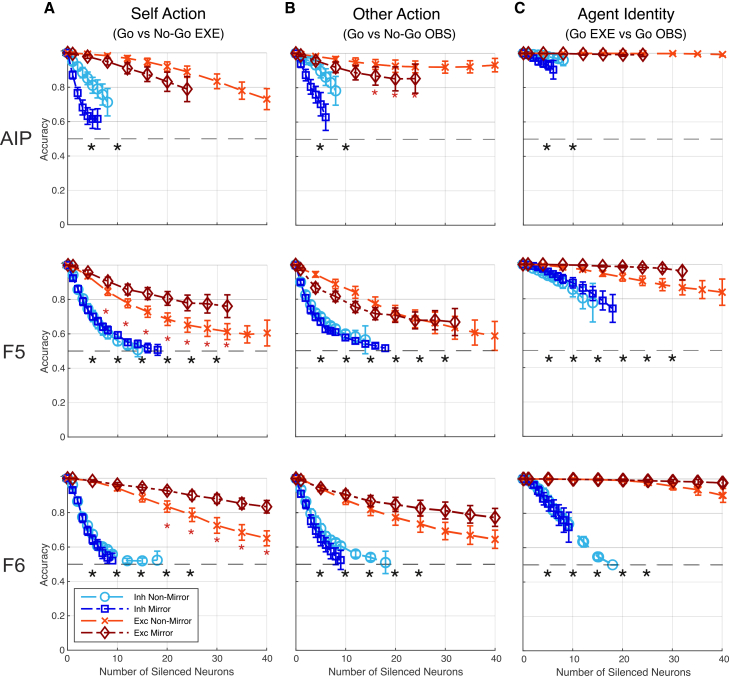
Silencing effects across areas Classifier accuracy for (A) self-action, (B) other-action, and (C) agent identity decoding as a function of the number of silenced neurons within each neuronal subgroup (categorized by cell type and functional class), shown separately for AIP (top), F5 (middle), and F6 (bottom). Data represent means across 20 RNNs and 50 random silencing samples per network; error bars indicate 95% confidence intervals across networks. Black asterisks mark significant differences (permutation test, *p* ≤ 0.05) between inhibitory and excitatory neurons regardless of mirror classification, and red asterisks mark significant differences between excitatory mirror and non-mirror neurons.

Overall (Figure 3), silencing inhibitory neurons (blue curves) had a much stronger impact on the discrimination between Go and No-Go conditions than silencing excitatory neurons (red curves) in all areas, during both action execution (“self action decoding”; Figure 3A) and observation (“other action decoding”; Figure 3B), in line with the well-established role of inhibitory neurons in shaping cognitive and motor processes^44^^,^^45^ (*p* < 0.01 in each area after silencing at least *N* = 5 excitatory or inhibitory neurons; permutation test).

Since inhibitory neurons account for approximately 20% of the total neuronal population in each area, we additionally expressed silencing strength as a percentage of each subpopulation, obtaining a pattern of results consistent with that observed using absolute neuron counts (Figure S4), indicating that the effect of inhibitory neuron silencing cannot be accounted for by their relatively smaller number.

Silencing excitatory neurons in areas F5 and F6 had a stronger impact on the classifier’s ability to decode self and other’s actions compared with silencing the same numbers of excitatory neurons in AIP (Figure S8). In particular, silencing excitatory non-mirror neurons in areas F5 and F6 impaired self-action decoding more strongly than silencing excitatory MNs (Figure 3A)(*p* ≤0.04 after silencing at least *N* = 8 excitatory mirror or non-mirror neurons in F5 and at least *N* = 20 in F6), whereas the same comparison was not significant for other-action decoding. In contrast, silencing excitatory MNs in AIP impaired other-action decoding more strongly than silencing excitatory non-mirror neurons in the same area (Figure 3B) (*p* = 0.01 after silencing at least *N* = 16 excitatory mirror or non-mirror neurons).

To explicitly test whether mirror/non-mirror silencing effects differed between execution and observation, we performed a complementary linear mixed-effects (LME) analysis (Figure S9; Table S5).This context dependence was quantified as the average observation-minus-execution difference in the mirror/non-mirror gap across the silencing curve. In excitatory populations, the resulting effect was prominent in F5 (*p* = 5.63 × 10^−18^), significant in F6 (*p* = 0.0017), and not significant in AIP (*p* = 0.376).

Since both tasks involved objects of three different sizes and shapes (i.e., a small cone, a big cone, and a ring), each to be grasped with a specific type of grip (i.e., a precision grip, a whole hand grip, and a hook grip), we also quantified the ability of the model to discriminate among the three objects in the execution (“self object decoding”) and observation (“other object decoding”) contexts (Figures S5, S6, and S7) as a function of the number of silenced units. The results showed a stronger decline in object decoding accuracy in the observation context relative to the execution context, regardless of the cell type or functional class of the inactivated neurons. This finding supports the greater relevance of information about the target object during action execution compared with observation. Interestingly, by training the classifier to discriminate among the three objects using data from the execution context and testing its performance on data from the observation context (and vice versa), we found that cross-context decoding remained at chance level in both cases (0.33 for three objects), suggesting a substantial remapping of object tuning across contexts rather than a shared coding scheme.^46^^,^^47^^,^^48^

Another long-standing issue in the MN literature concerns whether, and to what extent, the shared coding of self and other’s actions still allows the system to distinguish the acting agent.^49^^,^^50^ We addressed this question by training our classifier to discriminate between self and other Go trials (“agent identity decoding,” Figure 3C) and found that classification accuracy remained particularly high relative to all other decoding conditions, regardless of the silenced cell type.

AIP neurons, in particular, exhibited a very limited contribution to agent-based coding, independently of the targeted neuronal subpopulation. Similarly, silencing excitatory neurons in areas F5 and F6 (both mirror and non-mirror) did not affect agent discriminability, in striking contrast with the inactivation of inhibitory neurons (both mirror and non-mirror), especially in area F6 (Figure S8), indicating that silencing these latter cells produced a stronger impairment in agent-decoding performance within the model (*p* < 0.01 in each area after silencing at least *N* = 5 excitatory or inhibitory neurons).

## Discussion

In this work, we developed a biologically inspired RNN model that successfully reproduces single-neuron activity across three key areas of the macaque AON—AIP, F5, and F6—during the execution and observation of a Go/No-Go grasping task. Within the biological constraints we imposed on our model architecture, we uncovered a strong influence of inhibitory interneurons on network dynamics and agent identity coding, providing model-based insight into how excitatory and inhibitory neurons differentially contribute to orchestrating actions of self and others.

Previous studies that attempted to model MNs and the AON have remained largely theoretical or schematic in nature, typically focusing on single premotor^51^^,^^52^^,^^53^^,^^54^ or parietal^55^ nodes of the network and lacking anatomo-functional detail (such as cell type and anatomical specificities). As a consequence, these models have been limited in their ability to generate testable predictions. In contrast, modern RNN models of motor control are typically trained in a goal-driven fashion to generate specific outputs, such as muscle activity or kinematics, based on modeled input signals.^23^^,^^24^^,^^56^ While these architectures revealed how low-dimensional dynamics can generate complex movements, they are inherently unsuited to capturing the inner mechanisms of neuronal networks underlying covert behaviors, such as action observation. To overcome both these limitations, we built a multi-area model incorporating cell-type specificity, Dale’s principle,^18^^,^^32^^,^^57^^,^^58^ and a regionally constrained architecture of inhibitory and excitatory neurons^28^^,^^59^ to reconstruct empirical neural dynamics directly from electrophysiological recordings. This approach allowed us to model both overt action execution and others’ action observation and to perform causal in silico perturbations at single-neuron resolution.

From the structural point of view, it should be emphasized that the connectivity patterns emerging from the trained RNNs reflect functional interactions rather than real anatomical connections. Accordingly, the learned connectivity should not be interpreted as a reconstruction of anatomical connectivity, but rather as one functionally valid solution that remains compatible with established principles of local and inter-areal anatomical organization. Inhibitory neurons consistently exhibited higher connectivity than excitatory ones across all graph metrics, in line with previous *in vivo*^60^^,^^61^ and in silico^62^ studies. The presence of local clusters of inhibitory neurons, reflecting both the imposed local-connectivity constraint and training-dependent reorganization, is consistent with their lower proportion in cortical circuits relative to excitatory cells^40^^,^^63^ as well as with their role in stabilizing network dynamics^64^^,^^65^ and triggering action initiation.^45^^,^^66^ At the inter-areal level, AIP showed a predominantly efferent connectivity profile; although this contrasts with anatomical evidence indicating that cortico-cortical connections are usually bidirectional,^38^^,^^67^ it aligns with AIP’s functional role as a relay for information regarding the visual features of objects^68^^,^^69^^,^^70^ and other’s actions.^4^^,^^39^^,^^71^ Similarly, F5 exhibited a predominantly afferent connectivity profile and the highest overall connectivity among all areas, consistent with a central functional role in the representation and coordination of one’s own actions with those of others.^15^^,^^72^ Furthermore, we also looked at possible differences in connectivity patterns between mirror and non-mirror neurons and found that they were indistinguishable. This is consistent with accumulating anatomo-functional evidence over the past 15 years supporting the view that motor and the variety of visuomotor neurons in the premotor cortex are spatially intermingled and share fundamental preparatory motor functions,^73^^,^^74^^,^^75^ so that the recruitment of a potential motor action is achieved through the activation of largely overlapping neuronal populations. In this view, motor and MNs constitute different subcomponents of the same anatomo-functional mechanism underlying the sensorimotor processing of actions.^50^^,^^70^^,^^73^^,^^75^ This similarity also extended to outgoing inter-area connectivity, which showed no marked segregation between excitatory mirror and non-mirror populations (Table S4).

The connectivity between mirror and non-mirror neurons was similar, but silencing excitatory non-mirror neurons produced larger reductions in the network’s ability to decode self actions than silencing MNs, in both F5 and F6. By contrast, silencing excitatory MNs of area AIP had a stronger impact on other-action decoding than silencing non-mirror neurons in the same area. Despite the relatively small number of some subpopulations (e.g., inhibitory neurons in AIP), these findings suggest that action decoding was differentially sensitive to the perturbation of distinct neuronal subpopulations across cortical areas and decoders.

Another interesting outcome of our virtual silencing experiments is that inhibitory neurons in F5 and F6 appear to exert the strongest influence on agent-decoding performance. In fact, silencing even a large number of excitatory neurons in any of the three areas does not significantly impact the network'scapacity to specify who (monkey or experimenter) is acting, whereas the silencing of inhibitory (mirror and non-mirror) neurons in F5 and, particularly, F6, strongly impaired agent-decoding accuracy. This is in line with previous evidence indicating the crucial role of areas F5 and, particularly, F6 in agent-based sensorimotor processes,^50^^,^^73^^,^^76^ self and other’s action monitoring^72^ and social coordination.^77^

By combining biological realism with targeted in silico perturbations, our approach provides a powerful tool to probe the functional organization of complex cortical networks and to generate experimentally testable predictions—particularly in systems where direct manipulation remains unfeasible. Expanding the model to incorporate findings from additional cortical^78^ and subcortical^3^^,^^8^ regions will pave the way toward a comprehensive understanding of the functional organization of the extended AON.

### Limitations of the study

While our biologically constrained RNN successfully reproduces empirical firing-rate dynamics, the current model focuses on a limited set of cortical nodes, namely AIP, F5, and F6. The AON is highly distributed, and future extensions could incorporate additional regions involved in action execution, observation, and sensorimotor control, including primary motor cortex,^79^ dorsal premotor cortex,^47^ prefrontal areas,^80^ and subcortical structures such as the putamen.^81^ Second, our classification of excitatory and inhibitory neurons relies on extracellular spike waveform features. Although informative, this approach does not provide direct neurochemical confirmation and cannot resolve the diversity of inhibitory interneuron subtypes. Finally, our framework lacks a direct behavioral readout, such as limb kinematics or muscle activity. Because the model does not explicitly generate overt motor outputs, the functional impact of in silico perturbations was evaluated indirectly through the performance of a neural decoder. While this strategy effectively tracks shifts in population-level representations, it prevents a direct mapping between targeted network perturbations and trial-by-trial behavioral changes. Linking these recurrent dynamics to downstream, measurable behavioral outcome represents an important next step for future modeling efforts.

## Resource availability

### Lead contact

Requests for further information and resources should be directed to and will be fulfilled by the lead contact, Luca Bonini (luca.bonini@unipr.it).

### Materials availability

This study did not generate new materials.

### Data and code availability

• The experimental dataset used for model training and validation is publicly available in a work by Tili et al.^29^
• Custom code used to implement, train, and simulate the RNN model in Python using TensorFlow is publicly available on GitHub: https://github.com/lucaguglielmi97/AON_RNN.
• Any additional information required to reanalyze the data reported in this paper is available from the lead contact upon request.

## Acknowledgments

This work was supported by Ministry of University and Research (MUR), National Recovery and Resilience Plan (NRRP), project MNESYS (PE0000006) “A multiscale integrated approach to the study of the nervous system in health and disease” (DN. 1553 11.10.2022 to R.B. and L.B.); European Research Council (ERC) StG-2015 grant WIRELESS (678307 to L.B.); ERC CoG-2020 grant EMACTIVE (101002704 to L.B.); and grant MUR “CIRCEM” (R20NJ7BBA7 to L.B.).

## Author contributions

R.B., L.B., and D.A. designed the research; L.G., D.A., A.V., and R.B. contributed new analytic tools; L.G. analyzed the data; L.G., R.B., and L.B. wrote the paper; all authors reviewed the paper.

## Declaration of interests

The authors declare no competing interests.

## STAR★Methods

### Key resources table

**Table:** 

REAGENT or RESOURCE	SOURCE	IDENTIFIER
**Deposited data**		

Previously published macaque AON single-neuron dataset	Tili et al.^29^	https://doi.org/10.1038/s41597-025-05299-9

**Software and algorithms**		

Custom TensorFlow 2 code for RNN modeling	This paper	GitHub: https://github.com/lucaguglielmi97/AON_RNN
Python	Python Software Foundation	https://www.python.org/; RRID: SCR_008394
TensorFlow	TensorFlow developers	https://www.tensorflow.org/; RRID: SCR_016345
MATLAB R2024a	MathWorks	https://www.mathworks.com/products/matlab.html; RRID: SCR_001622

### Method details

#### Experimental data and behavioral paradigm

The neural activity of 355 single units within AIP, F5, and F6 was recorded while monkeys performed a Go/No-Go grasping task and observed an experimenter performing the same task.^15^ Both the execution and observation tasks followed the same temporal structure, beginning with an auditory cue conveying the instruction: a high-frequency tone signaled the Go condition, prompting the monkey to execute a grasping action, whereas a low-frequency tone indicated the No-Go condition, requiring the monkey to withhold movement and maintain fixation. In both conditions, light was turned on and the monkeys were presented with one of three different objects, each requiring a distinct grip type: a ring, necessitating a hook grip, a small cone, requiring a precision grip, and a large cone, demanding whole-hand prehension. After the sound ceased, the monkey either reached to grasp and pull the object to obtain a reward (Go) or maintained fixation without moving (No-Go). In the observation context, an experimenter executed the same task while the monkey observed the actions from a 90° visual perspective.

The dataset thus comprised firing-rate time series for each of the 355 neurons across the 12 task conditions (3 objects × 2 Go/No-Go × 2 execution/observation), with 10 trials recorded per condition. All neurons were pooled across the three monkeys for analysis. Firing rates were computed for each neuron, averaged across trials, and smoothed with a 60 ms Gaussian kernel. Activity was aligned to two task events—object presentation (-1.3 s–0.7 s relative to onset) and the Go/No-Go signal (-0.3 s–1.2 s relative to onset)—following the same procedure as previously described.^15^ For model training, the two aligned epochs were concatenated to form a single 3.5 s trajectory per condition.

Mirror neurons were identified by testing firing-rate modulation during the movement epoch in both execution and observation contexts using a sliding *t* test (window = 200 ms, step = 20 ms, *p* < 0.05, uncorrected). Neurons showing significant modulation for at least five consecutive windows (≥300 ms) in both contexts were classified as mirror, thereby reducing the likelihood of spurious classifications driven by isolated significant windows, whereas neurons significant only during execution were classified as motor neurons.

Neurons were further classified as excitatory or inhibitory according to their extracellular spike waveform characteristics, by clustering trough-to-peak duration and repolarization time parameters.^82^

Finally, firing rates were normalized by dividing each neuron’s activity by the global maximum observed across all neurons and conditions, yielding values in the range [0, 1] consistent with the rate-based model.

#### Rate model and input design

A wide range of dynamical systems have been employed to model population-level neural activity, including spiking networks, integrate-and-fire neurons, and coarse-grained rate-based formalisms.^83^^,^^84^^,^^85^^,^^86^ Among these approaches, rate-based models describe neuronal activity through continuous variables evolving under recurrent interactions, providing a tractable and analytically accessible framework for studying high-dimensional nonlinear systems. These models have been instrumental in revealing core dynamical regimes of cortical circuits, including fixed-point attractors, chaotic fluctuations, and stimulus-driven trajectories relevant to memory and decision-making.^30^^,^^83^^,^^87^

Continuous rate RNNs extend this framework and are widely adopted in neuroscience and machine learning as flexible models for temporal computation, owing to their differentiable dynamics that allow efficient optimization via gradient-based methods. This makes them particularly suited for reproducing large-scale neural activity from experimental data.^31^

Here, we employed a continuous rate RNN with *N* = 355 units, matching the number of recorded neurons in the experimental dataset. Each model unit was mapped one-to-one to a neuron from the experimental data. The dynamics of the system are governed by a set of equations (Equation 1), and the firing rate *r*_*i*_ of unit *i* is obtained by applying a standard sigmoid function to its synaptic current variable, ensuring that the values are within the range [0,1]:

(Equation 4) $$\phi \left(x_{i}\right)=\frac{1}{1+e^{-x_{i}}}.$$

The external current *I*_*ext*_ includes Gaussian white noise, a neuron-specific background current $\theta \in R^{N\times 1}$, and task-specific input signals modeling the visual and auditory stimuli presented during the experiment:

(Equation 5) $$I_{\mathrm{ext}}=N\left(0,0.1^{2}\right)+\theta +W_{in}\;\cdot \;u,$$

where $u\in R^{N_{in}\times T}$ is a collection of *N*_*in*_ time-varying stimulus signals entering the network through the input weight matrix $W_{in}\in R^{N\times N_{in}}$. Each input weight could assume positive or negative values, thereby enhancing or inhibiting the target neuron’s activity. The Gaussian noise term $N\left(0,0.1^{2}\right)$ had zero mean and a standard deviation of 0.1, introducing small fluctuations in neuronal activity to mimic the intrinsic variability observed in cortical firing patterns. The background current *θ* was adjusted such that each neuron’s baseline firing rate (i.e., when *x*_*i*_ = 0) matched the experimentally observed mean baseline.

The experimental stimuli were distinguished between visual and auditory. Three distinct visual stimuli were presented, corresponding to the three different objects, while two auditory stimuli differentiated between the Go and No-Go conditions. Visual stimuli were parameterized as step functions that increased from 0 to 1 upon light onset and remained active until task completion, whereas auditory stimuli were step functions that rose to 1 during sound presentation and returned to 0 after their cessation. A small delay of 0.1 s was considered for these signals compared to the experimental triggers. Visual inputs targeted neurons in AIP, auditory inputs those in F6, while both types were delivered to F5 at half amplitude (0.5). Specifically, in each of the six combinations involving the 3 objects and the 2 conditions, a visual stimulus targeted AIP and an acoustic stimulus was directed to F6, along with the corresponding stimuli directed to F5. The same stimuli structure was used for both execution and observation contexts, but two distinct sets of input weights were optimized separately to differentiate between tasks performed in these two situations: $W_{in}=W_{in}^{\mathrm{EXE}}$ or $W_{in}=W_{in}^{\mathrm{OBS}}$. This design implies that part of the execution/observation differentiation is introduced at the level of external inputs. However, supplementary analysis of the trained dynamics in the absence of input and noise indicates that context-dependent structure is also retained by the recurrent dynamics of the model (see Figure S10).

#### Training details

The directed connectivity matrix *W* was initialized as a fully connected matrix, with each element sampled from a Gaussian distribution with 0 mean and variance $\frac{g^{2}}{N}$, where *N* is the number of neurons and *g* is a parameter set to *g* = 1.5. Self-connections were prevented by setting the diagonal of *W* to zero.

Following previous work,^88^ Dale’s principle was enforced directly at initialization by constraining each unit to emit either exclusively excitatory or exclusively inhibitory outputs. Neurons in our model were classified as excitatory or inhibitory based on empirical spike-shape analyses,^15^ unlike most modeling studies, in which this information is typically unavailable and a fixed percentage (e.g., 20%) of neurons is randomly labeled as inhibitory.^88^ This empirically grounded classification ensures biologically valid sign constraints on synaptic weights and helps maintain excitation–inhibition balance and stabilize network dynamics.^89^

Additionally, in our model inhibitory connections were restricted exclusively among neurons belonging to the same area, meaning that for inhibitory neurons *j* the links *w*_*ij*_ were set to zero when *i* and *j* did not belong to the same anatomical region. This constraint is consistent with neurophysiological evidence that short-range inhibition dominates local circuit control and contributes to the functional segregation of cortical modules.^33^^,^^90^ Apart from this restriction and the removal of self-connections, no other structural priors were imposed on *W*, allowing the recurrent architecture to develop freely through training and to capture emergent connectivity patterns driven solely by the requirement to reproduce experimental neural activity.

The network equations (Equations 1 and 2) were integrated using the first-order Euler method with a time step Δ*t* = 5 ms, simulating the firing rate dynamics of the network under each of the 12 experimental conditions. Experimental firing rates $\hat{r}$ were obtained as trial-averaged and smoothed trajectories (10 trials per condition; 60 ms Gaussian kernel) and served as training targets.

The RNN was trained to reproduce these empirical firing-rate dynamics by minimizing the root mean squared deviation between simulated (*r*) and experimental $\left(\hat{r}\right)$ activity:

(Equation 6) $$L=\sqrt{\overset{12}{\underset{\mathrm{task=1}}{\sum }}\overset{T}{\underset{t=1}{\sum }}\overset{N}{\underset{i=1}{\sum }}{\left(r_{i,t,task}-{\hat{r}\;}_{i,t,task}\right)}^{2}},$$

where *T* is the total length of the time series.

To minimize this loss function, backpropagation through time (BPTT) was used in combination with the adaptive moment estimation (Adam) stochastic gradient descent algorithm,^91^ as implemented in TensorFlow (Python). The learning rate was set to 0.01, while the decay rates of the first and second moments were kept at their default TensorFlow values. This procedure was utilized to optimize the neuron-specific synaptic decay time constants $\tau_{i}^{d}$, the recurrent connectivity matrix *W*, and the sets of input weights $W_{in}^{\mathrm{EXE}}$ and $W_{in}^{\mathrm{OBS}}$, the latter being initialized from a Gaussian random matrix with 0 mean and unit variance.

A regularization term in the form of L2 norm has been incorporated into the loss function for the connectivity matrix and the input weight sets to prevent them from assuming biologically unrealistic values:

(Equation 7) $$L_{\mathrm{Reg}}=L+\lambda \cdot \left(\|W{\|}_{F}+\|W_{in}^{\mathrm{EXE}}{\|}_{F}+\|W_{in}^{\mathrm{OBS}}{\|}_{F}\right),$$

where in our case *λ* = 0.01 and

(Equation 8) $$\|A{\|}_{F}=\sqrt{\overset{M}{\underset{i=1}{\sum }}\overset{N}{\underset{j=1}{\sum }}|a_{ij}{|}^{2}}$$

denotes the Frobenius norm of the *M* × *N* matrix *A* whose elements are *a*_*ij*_. This regularization penalizes extreme weight magnitudes, discouraging overfitting and promoting stable, biologically plausible connectivity.^63^^,^^92^

The synaptic decay time constants $\tau_{i}^{d}$ were bounded within a biologically plausible range, following previous work.^32^ Specifically, they were constrained between a minimum value $\tau_{\text{min}}^{d}=4$ ms and a maximum value $\tau_{\text{max}}^{d}=20$ ms, with a total range $\tau_{\text{step}}=\tau_{\text{max}}^{d}-\tau_{\text{min}}^{d}=16$ ms. To ensure positive values and smooth distribution across neurons, each $\tau_{i}^{d}$ was parameterized as:

(Equation 9) $$\tau_{i}^{d}=\tau_{\text{min}}^{d}+\tau_{\text{step}}\;\cdot \;\sigma \left(N\left(0,1\right)\right),$$

where *σ*(*x*) denotes the sigmoid function.

Post-training analyses of the optimized synaptic decay constants did not reveal significant differences across neuronal classes. A detailed analysis of the learned input weights, including direct comparisons between execution- and observation-related input matrices, is reported in the Supplemental Materials and briefly summarized in the main Results.

Due to the high dimensionality of the parameter space, we independently trained 20 networks with distinct random initializations to evaluate the robustness and generalizability of the inferred connectivity patterns. All networks were trained on the full dataset using the same optimization procedure described above and consistently reproduced the experimental neural activity with high accuracy (Table S1).

In addition to the main training procedure on all tasks, we performed separate training runs exclusively on execution-related and observation-related tasks to assess the robustness of the network architecture. This analysis confirmed that the connectivity patterns remained consistent across conditions and that key features were preserved under both training regimes (Figures S2E and S2F).

#### Connectivity metrics

Following training, we analyzed the structure of each recurrent connectivity matrix *W* by quantifying key network features. The *W* matrices were first binarized by setting all nonzero weights to 1, thereby preserving the full learned connectivity structure without imposing arbitrary thresholds.

We then computed the percentage of active synaptic connections within and between anatomical areas, defined as the fraction of nonzero connections relative to all possible ones. When computing connection probabilities, inhibitory projections that had been set to zero by design (i.e., inter-areal inhibitory connections) were excluded from the denominator, ensuring that the resulting values reflected only connections that were allowed to form during training. Connectivity percentages were computed for each of the 20 independently trained networks and then averaged across models to ensure robustness and generalizability. Variability across networks is reported as standard deviation, which remained consistently low, indicating high reliability of the measured features (Figure 2; Tables S2 and S3).

We also computed the asymmetry index (Equation 3) for each area to quantify the imbalance between efferent and afferent projections, providing a complementary characterization of inter-areal connectivity patterns.

To analyze the topological role of individual neurons, we computed two node-level metrics: local efficiency and betweenness centrality. Local efficiency quantifies the degree of connectivity among a neuron’s immediate neighbors (i.e., neurons directly connected by at least one nonzero synaptic weight, irrespective of its strength). It reflects the local clustering and resilience of the network and it is mathematically defined as^36^:

(Equation 10) $$E_{\text{loc}}\left(i\right)=\frac{1}{k_{i}\left(k_{i}-1\right)}\underset{\begin{array}{c} j,k\in N_{i}\\ j\neq k \end{array}}{\sum }\frac{1}{d_{jk}\left(i\right)},$$

where *N*_*i*_ is the set of neighbors of neuron *i*, *k*_*i*_ is the number of neighbors, and *d*_*jk*_(*i*) is the shortest path distance between neurons *j* and *k* in the subgraph obtained by removing neuron *i* (i.e., considering only paths that do not go through *i*).

Betweenness centrality measures the extent to which a neuron acts as a bridge on the shortest paths between pairs of other neurons, highlighting its potential role as a communication hub:

(Equation 11) $$BC\left(i\right)=\underset{\begin{array}{c} s\neq i\neq t \end{array}}{\sum }\frac{\sigma_{st}\left(i\right)}{\sigma_{st}},$$

where *σ*_*st*_ is the total number of shortest paths between nodes *s* and *t*, and *σ*_*st*_(*i*) is the number of those paths that pass through node *i*. These metrics were computed for each neuron in all trained networks and averaged across the 20 models. The resulting variability, measured as the standard deviation across models, was minimal, supporting the consistency of the observed patterns.

Neurons were grouped by (i) cell type (excitatory or inhibitory), (ii) functional identity (mirror or non-mirror), and (iii) anatomical area (AIP, F5, or F6). Group-wise comparisons of connectivity metrics were performed using two-sided Mann–Whitney U tests, treating each neuron as an independent statistical unit. Because data distributions were not assumed to be normal, non-parametric tests were used throughout. No correction for multiple comparisons was applied. These tests were applied to predefined groupwise contrasts within each analysis, rather than as a global screen across all possible combinations, and the reported *p*-values should be interpreted in that comparison-specific context.

For each neuron, the metric of interest was averaged across the 20 trained networks before performing group comparisons, ensuring that statistical inference reflected population-level rather than model-specific variability.

To assess the presence of homophily, i.e., a preference for neurons to connect with others of the same type, we computed Newman’s assortativity coefficient, defined as^42^:

(Equation 12) $$r=\frac{\sum_{i}e_{ii}-\sum_{i}a_{i}b_{i}}{1-\sum_{i}a_{i}b_{i}},$$

where *e*_*ij*_ is the fraction of edges that connect a node of type *i* to a node of type *j*, *a*_*i*_ = *∑*_*j*_*e*_*ij*_ is the fraction of edges connected to nodes of type *i* at the source end, and *b*_*j*_ = *∑*_*i*_*e*_*ij*_ is the fraction of edges connected to nodes of type *j* at the target end. A positive value of *r* indicates assortative mixing (i.e., a tendency to connect to same-type nodes), while a negative value reflects disassortative mixing (i.e., a preference for cross-type connections). A value close to zero suggests random mixing.

#### Synthetic activity classification

To assess how perturbations affect task-relevant neural representations, we trained a set of classifiers to decode specific behavioral variables from synthetic neural activity generated by our models. Each classifier targeted a distinct aspect of task representation. In this framework, classifier performance was used as an operational readout of how strongly task-relevant information remained represented at the population level in the synthetic network activity. One classifier performed “agent identity” decoding, distinguishing between Go trials executed by the agent and those observed. Two classifiers performed action decoding, differentiating between Go and No-Go trials separately in the execution (“self action decoding”) and observation (“other action decoding”) contexts. Finally, two classifiers decoded object identity during Go trials, again separately for execution (“self object decoding”) and observation (“other object decoding”) conditions.

To perform these decoding tasks, we employed bidirectional Long Short-Term Memory classifiers. This architecture is particularly suited to neural time series, as it integrates information from both preceding and subsequent activity to capture temporally asymmetric dynamics in population responses.^93^^,^^94^^,^^95^

All classifiers were trained and evaluated on synthetic firing rate sequences generated from the 20 independently trained RNNs, since the empirical neural recordings provided only one trial-averaged trajectory per condition—insufficient for robust classifier training. To ensure statistical reliability and generalization, synthetic datasets were generated by varying Gaussian noise and initial conditions across multiple runs (≥100 per network instance), and subsequently split 80%/20% into training and testing sets. This procedure produced hundreds of trajectories per condition and yielded stable decoder performance across networks.

Each classifier was trained on a fixed 0.8-s window immediately after the end of the Go/No-Go signal, corresponding to the period in which the monkey executed or withheld the movement. This temporal window corresponds to the epoch of strongest task-related modulation observed experimentally and was kept identical across all models and conditions.

After training, classifiers were used to test whether task-relevant distinctions could still be decoded after in silico silencing of selected neuronal populations. To isolate the effects of altered network dynamics from trivial signal loss, we performed an additional control analysis in which neuronal activity was set to zero directly at the classifier input—without re-running the RNN. This decoder-only ablation approach did not degrade classification accuracy, even when up to 50 neurons were zeroed out, confirming that the performance reductions observed during in silico silencing reflected genuine perturbations of the underlying network dynamics rather than simple information removal.

#### Neuron silencing procedure

To investigate how different neuronal subpopulations contribute to task-related processing, we systematically inactivated neurons in our RNNs and analyzed the impact on classifier performance. Accordingly, changes in classifier performance were interpreted as reflecting how silencing affected the population-level representation of task-relevant information in the network. Neuron silencing was implemented by setting the firing rate of selected neurons to zero for the entire analyzed time window. This ensured that the silenced neurons no longer influenced the network’s activity, allowing us to assess how their absence affected overall network dynamics and task-related representations.

We specifically aimed to investigate the role of distinct neuronal subpopulations within each of the three cortical areas, considering both their type—excitatory or inhibitory—and their classification as mirror or non-mirror neurons. To this end, classifier accuracy was evaluated as a function of the number of silenced neurons in each subpopulation, allowing us to assess how progressive inactivation altered task-related decoding performance. Accuracy values are reported on an absolute scale between 0 and 1, with the unperturbed network (0 silenced neurons) corresponding to perfect decoder performance (accuracy = 1). Each subsequent point therefore directly reflects the fraction of correctly decoded trials at the corresponding level of silencing.

To address the variability in neuron selection, we generated 50 independent random subsets of the number of neurons to be silenced for each subpopulation. For each subset, we applied the silencing procedure and computed the classifier accuracy, with the final results obtained by averaging across these 50 selections. Additionally, since the analysis was conducted across 20 independently trained RNNs, we further averaged the results across networks. Through this approach, we obtained the classification accuracy trends as a function of the number of silenced neurons for each neuronal subclass, separately for the three cortical areas.

Given that the neuronal subpopulations vary in size—for instance, the inhibitory mirror neurons in AIP constitute the smallest subpopulation with only 6 neurons, while the excitatory non-mirror neurons in F6 represent the largest with 70 neurons—we also implemented a percentage-based silencing approach. This method involved silencing a fixed proportion of neurons from each subpopulation, rather than a fixed number. By doing so, we ensured fair comparisons across the different groups, mitigating the impact of discrepancies in the total number of neurons within each class. Absolute (fixed-number) and percentage-based silencing provide complementary perspectives: the former reflects manipulations targeting a fixed set of neurons, as in typical experimental interventions, whereas the latter captures how each subpopulation’s contribution scales with its relative size. Importantly, both approaches yielded consistent qualitative results, confirming the robustness of our findings for both the action decoder (Figures 3 and S4) and the object decoder (Figures S5 and S6).

### Quantification and statistical analysis

#### Statistical analysis and permutation tests

To assess the statistical significance of differences in classifier performance when silencing different neuron subpopulations, we employed pairwise permutation tests. For each comparison, we performed 100 permutation iterations. In each iteration, neuron labels were shuffled randomly to generate artificial groups of the same size but with mixed identities. Within these shuffled groups, we randomly selected 50 subsets of neurons to silence by setting their firing rates to zero, and computed the classifier accuracy for each subset. The accuracy values from these 50 subsets were averaged to obtain the performance estimate for each shuffled group. This procedure was performed independently for each of the 20 trained networks, and, for each permutation iteration, accuracies were averaged across networks to obtain a single group-level value.

Repeating this process 100 times yielded a pooled null distribution describing the expected variability of mean accuracy differences across independently trained RNNs. The observed accuracy difference between the original (non-shuffled) groups was then compared against this distribution to compute a *p*-value, defined as the fraction of permutations producing a difference as large as or larger than the observed one. In this framework, the *p*-value therefore reflects the probability of observing a consistent group-level effect across models under the null hypothesis that group identity does not influence silencing outcomes. Statistical significance was set at *p* ≤ 0.05. Across silencing levels, permutation tests were therefore used to identify the range of perturbation strengths at which differences between predefined neuronal groups became detectable, rather than to support a single omnibus inference across all levels of silencing. Importantly, these permutation-based comparisons were performed within each decoder/context and were not intended to test whether mirror/non-mirror differences formally interacted with action context.

To complement the permutation analysis, 95% confidence intervals for classifier accuracy were computed across the 20 networks, providing an additional measure of reliability across models and ensuring that results were not driven by individual network variability.

Permutation tests were performed for both absolute and percentage-based silencing procedures. In the absolute approach, a fixed number of neurons (typically between 5 and 40 per group, depending on population size) were inactivated. In the percentage-based approach, a constant fraction (starting from 35%) of each subpopulation was silenced. Applying the same permutation framework to both methods allowed us to verify that the main effects were consistent regardless of the silencing criterion.

Classifier performance was compared after silencing different neuron subpopulations, with all analyses conducted separately for each of the three cortical areas. Specifically, we tested for differences between mirror and non-mirror neurons within excitatory and inhibitory populations independently. We also compared the effects of silencing inhibitory versus excitatory neurons, irrespective of functional classification. In addition, each of the four subpopulations—excitatory mirror (AIP = 27, F5 = 33, F6 = 66), inhibitory mirror (AIP = 6, F5 = 19, F6 = 9), excitatory non-mirror (AIP = 45, F5 = 40, F6 = 70), and inhibitory non-mirror (AIP = 8, F5 = 14, F6 = 18)—was analyzed independently to evaluate the effects of silencing across cortical areas.

A notable limitation arose in the case of AIP, where the small size of the inhibitory populations affected the reliability of the permutation tests. Specifically, the inhibitory mirror and non-mirror groups in AIP consisted of only 6 and 8 neurons, respectively. With such limited sample sizes, selecting even a small number of neurons (e.g., 5) per group often failed to capture sufficient variability, thereby compromising the robustness of the test. This constraint should be taken into account when interpreting the results involving these subpopulations, as the small number of neurons may affect the statistical power of the tests.

To directly assess whether mirror/non-mirror differences in action decoding depended on action context, we performed a complementary linear mixed-effects analysis. Models were fitted separately for each cortical area (AIP, F5, F6) and cell type (excitatory, inhibitory), using the formula:

$$Accuracy∼{NumSilenced}_{c}\times Context\times FunctionalClass+\left(1|Network\right).$$

Here, *NumSilenced*_c_ is the centered number of silenced neurons, *Context* indicates execution/self-action or observation/other-action decoding, and *Functional Class* refers to non-mirror and mirror neurons. Network identity was included as a random intercept to account for baseline differences across independently trained RNNs and repeated measurements from the same network.

Because *NumSilenced* was treated as a continuous covariate, the context dependence of mirror/non-mirror differences was summarized across the progressive-silencing curve. For each fitted model, we computed the LME-derived marginal interaction contrast

$$\Delta_{\text{int}}=\left[{\left(M-nM\right)}_{\text{OBS}}-{\left(M-nM\right)}_{\text{EXE}}\right],$$

averaged over the common silencing range from fixed-effect model predictions. This contrast quantified the average observation-minus-execution difference in the mirror/non-mirror gap across the silencing curve.

All statistical, connectivity, decoding, and in silico silencing analyses were implemented in MATLAB R2024a (MathWorks).

## Supplemental information

Document S1. Figures S1–S10 and Tables S1–S5, and Supplemental notes
