# Supplementary material for: Selective perturbation of mirror and non-mirror neurons in an in silico model of the action observation network
Source: iScience. 2026 Jul 17;29(8):116776. doi: 10.1016/j.isci.2026.116776 (PMC13401003; doi:10.1016/j.isci.2026.116776)
Supplement: Document S1. Figures S1–S10 and Tables S1–S5, and Supplemental notes [file mmc1.pdf]

iScience, Volume *29*

Supplemental information

Selective perturbation of mirror and non-mirror neurons in an in silico model of the action observation network

Luca Guglielmi, Davide Albertini, Alessandro Vezzani, Raffaella Burioni, and Luca Bonini

Supplemental information

Supplemental Figures

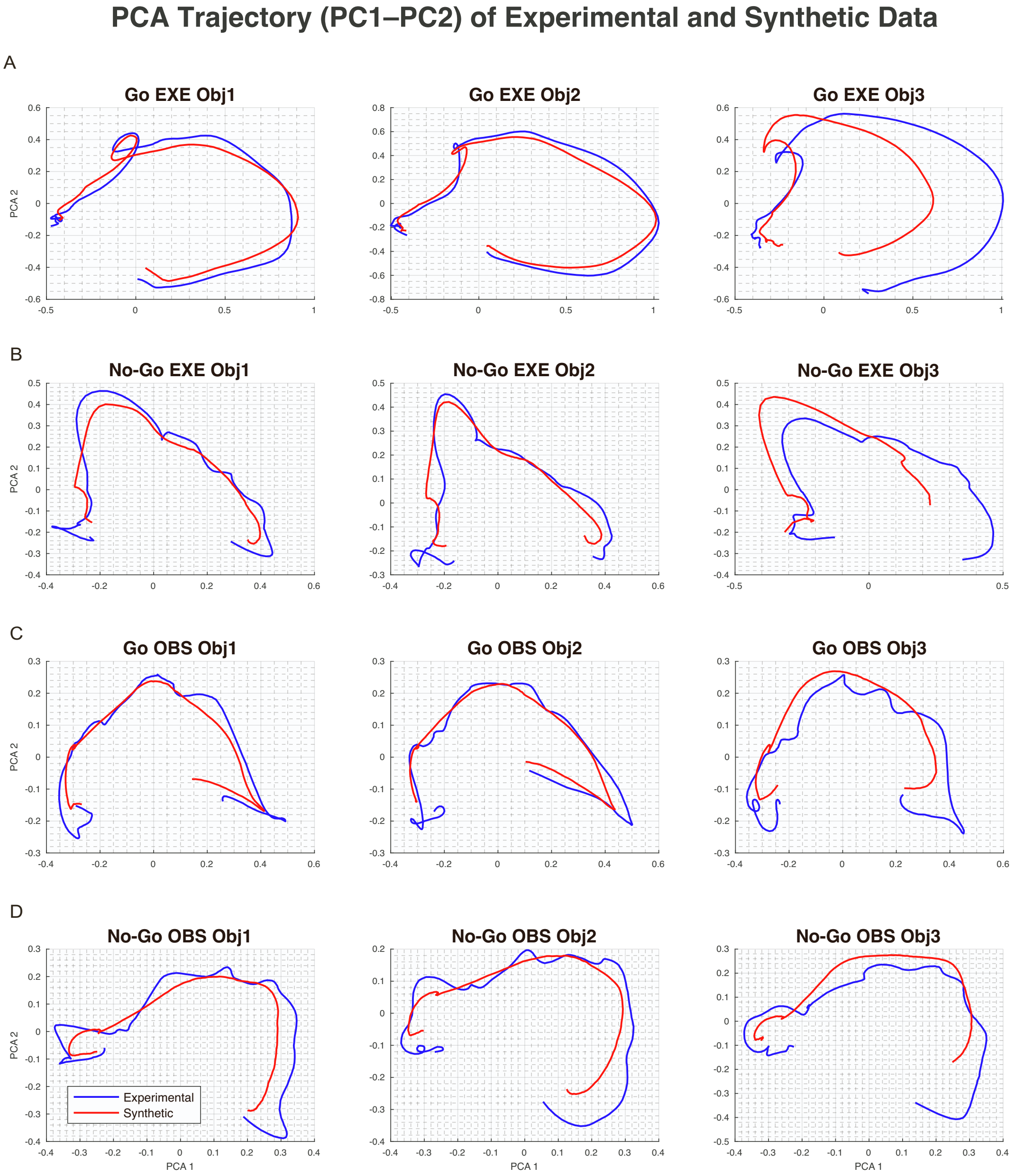

Supplemental Figure 1 | PCA comparison, related to Figure 1. Each subplot shows the PCA trajectory (PC1–PC2) of experimental (blue) and synthetic (red) firing rates for all the tasks. Rows (A–D) correspond to task conditions: (A) Go EXE, (B) No-Go EXE, (C) Go OBS, (D) No-Go OBS. Columns correspond to the three object identities (Obj1–Obj3).

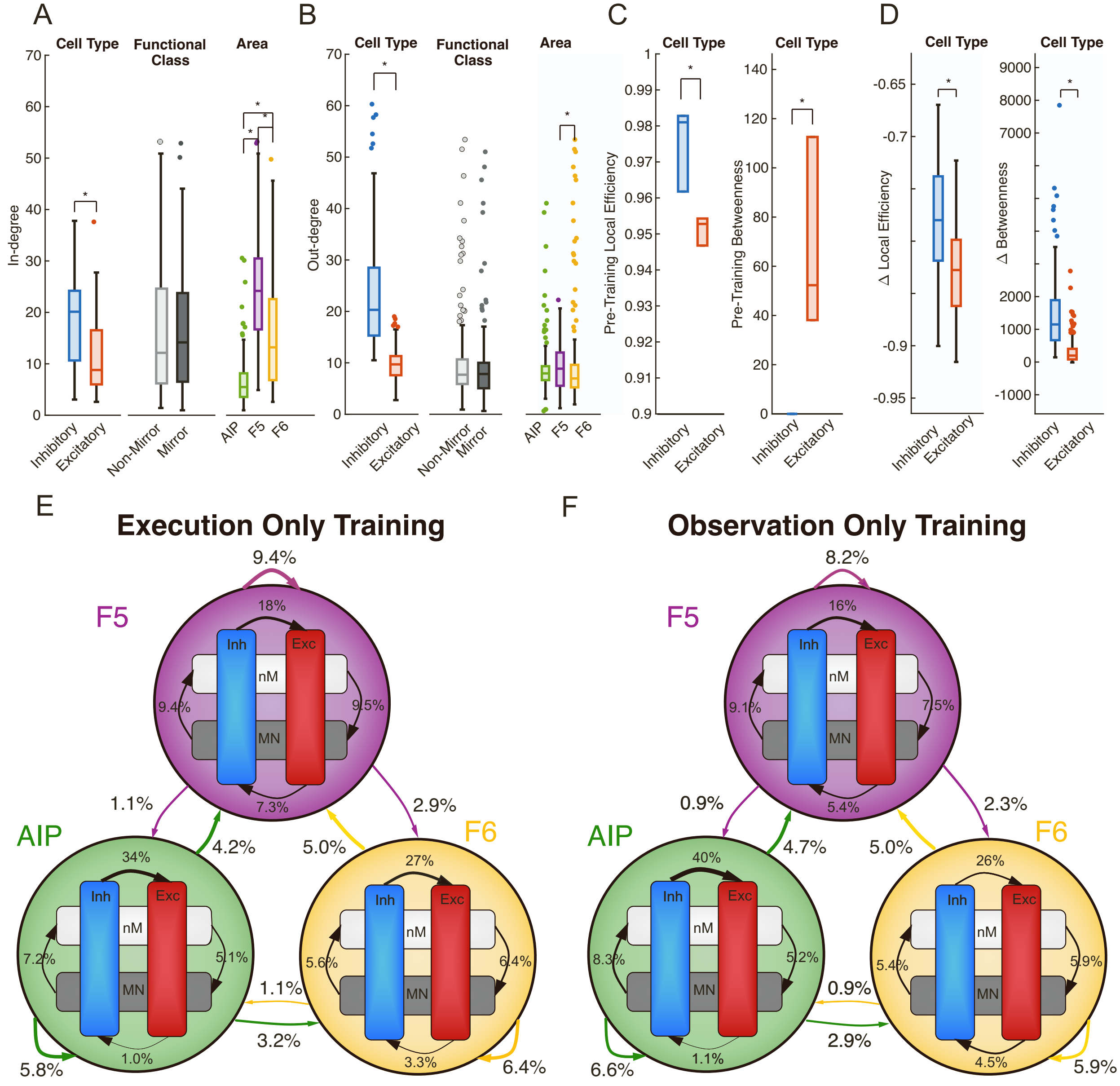

Supplemental Figure 2 | Connectivity patterns and degree distributions, related to Figure 2. (A) In-degree distributions for neurons grouped by cell type (excitatory, Exc; inhibitory, Inh), functional class (mirror, MN; non-mirror, nMN), and anatomical area (AIP, F5, F6). In each boxplot, the central line represents the median, the box spans the interquartile range (IQR, 25th–75th percentile), whiskers extend to data points within 1.5× the IQR, and outliers are shown as individual dots. Asterisks indicate significant differences ($p < 0.05$, Mann–Whitney U test) between the corresponding groups. (B) Same as in panel (A), but for out-degree distributions. (C) Pre-training distributions of local efficiency (left) and betweenness centrality (right), compared between excitatory and inhibitory neurons. (D) Training-induced changes in the same metrics, computed as the post-minus-pre training difference for each neuron. (E) Inter- and intra-area connectivity patterns among AIP, F5, and F6, subdivided by cell type (Exc, red; Inh, blue) and functional class (MN, dark shades; nMN, light shades), for RNNs trained exclusively on execution tasks. Percentages indicate mean connection probabilities across 10 network realizations. (F) Same as in panel (E), but for RNNs trained exclusively on observation tasks.

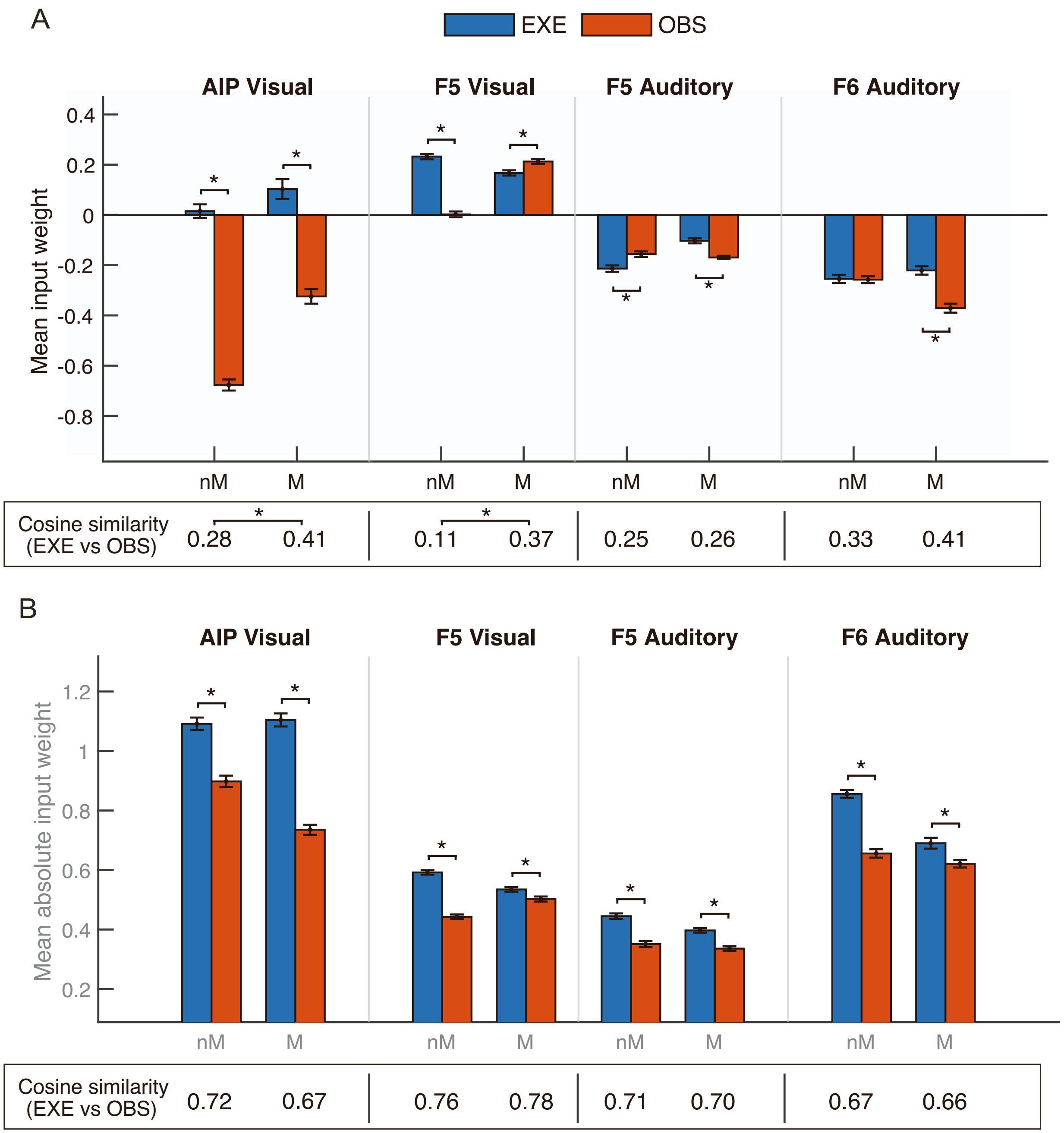

Supplemental Figure 3 | Context-dependent modulation of learned input weights, related to Figure 2. (A) Mean signed input weights (mean $\pm$ SEM across the 20 independently trained networks) for execution (EXE, blue) and observation (OBS, orange) conditions in neuronal populations grouped by anatomical area and functional class (mirror, M; non-mirror, nM). (B) Same analysis performed on the absolute values of the input weights. Visual inputs are shown for AIP and F5 (left), and auditory inputs for F5 and F6 (right). Asterisks above bars indicate significant differences ($p < 0.05$, paired Wilcoxon signed-rank test across networks) between EXE and OBS within the corresponding population. Values reported below each population indicate the cosine similarity between EXE and OBS input patterns across neurons within that population, computed separately for visual and auditory channels; asterisks above these values indicate significant differences between mirror and non-mirror populations.

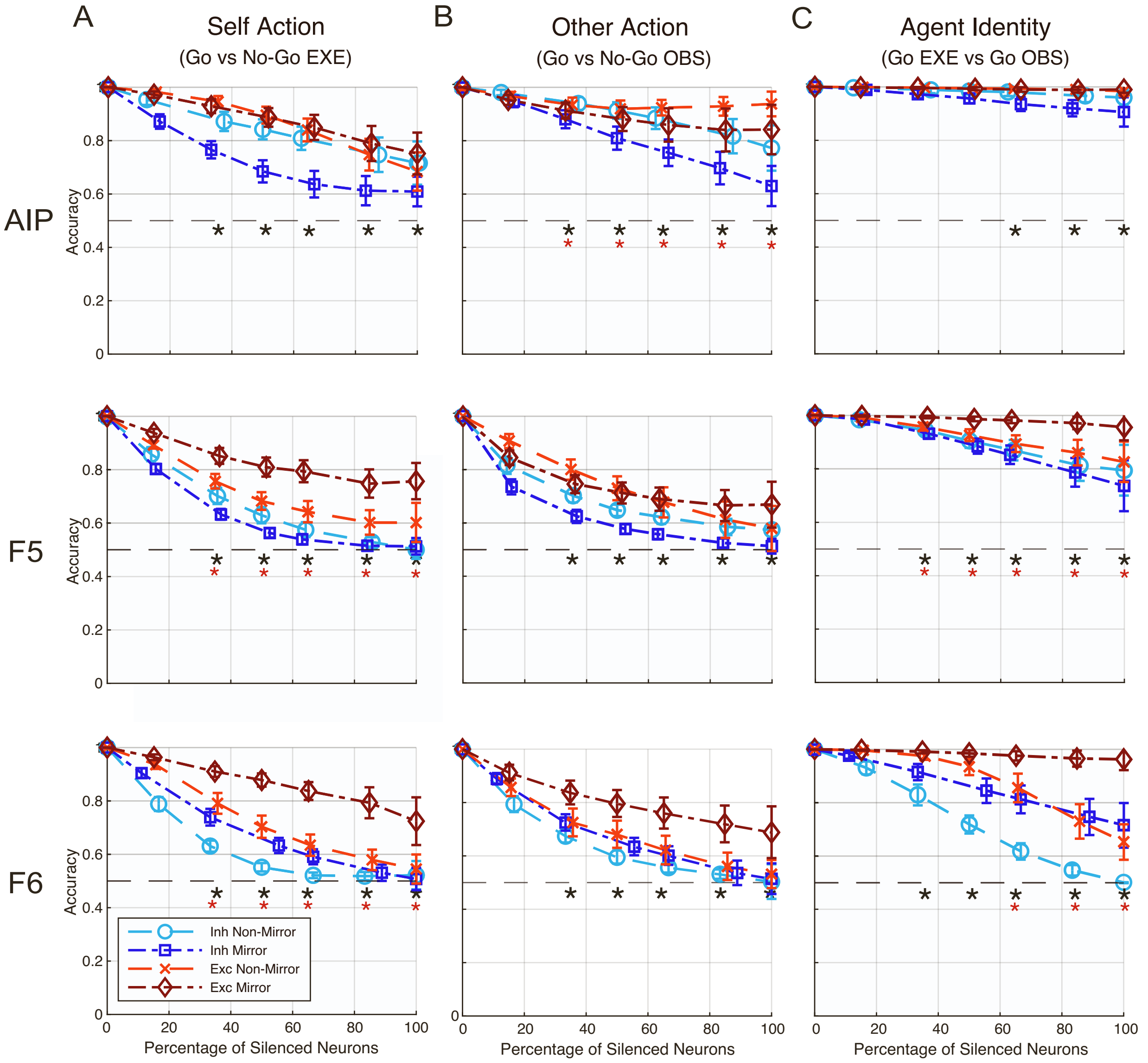

Supplemental Figure 4 | Percentage-based silencing effects across areas, related to Figure 3. Classifier accuracy for (A) self-action, (B) other-action, and (C) agent identity decoding as a function of the percentage of silenced neurons within each neuronal subgroup (categorized by cell type and functional class), shown separately for AIP (top), F5 (middle), and F6 (bottom). Data represent means across 20 RNNs and 50 random silencing samples per network; error bars indicate 95% confidence intervals across networks. Black asterisks mark significant differences (permutation test, $p \leq 0.05$) between inhibitory and excitatory neurons regardless of mirror classification, and red asterisks mark significant differences between excitatory mirror and non-mirror neurons.

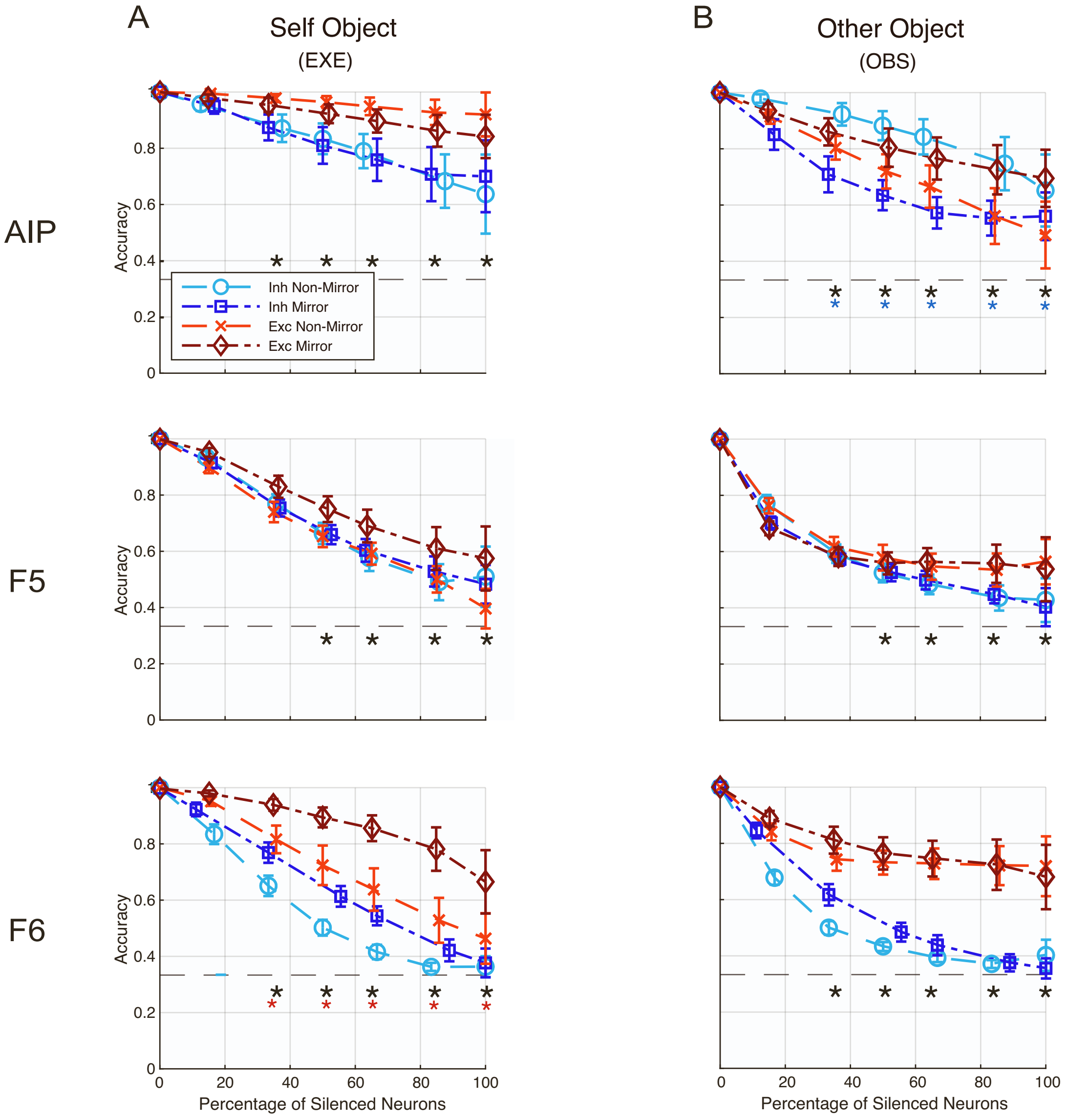

Supplemental Figure 5 | Percentage-based silencing effects across areas for object decoding during execution and observation, related to Figure 3. (A) Classifier accuracy for self-object and (B) other-object decoding as a function of the percentage of silenced neurons within each cell group, categorized by cell type—excitatory (Exc, red) and inhibitory (Inh, blue)—and functional class—mirror (MN, dark shades) and non-mirror (nM, light shades)—shown separately for AIP (top), F5 (middle), and F6 (bottom). Data represent means across 20 RNNs and 50 random silencing samples per network; error bars indicate 95% confidence intervals across networks. Black asterisks mark significant differences (permutation test, $p < 0.05$) between inhibitory and excitatory neurons regardless of functional class, and red asterisks mark significant differences between excitatory mirror and non-mirror neurons.

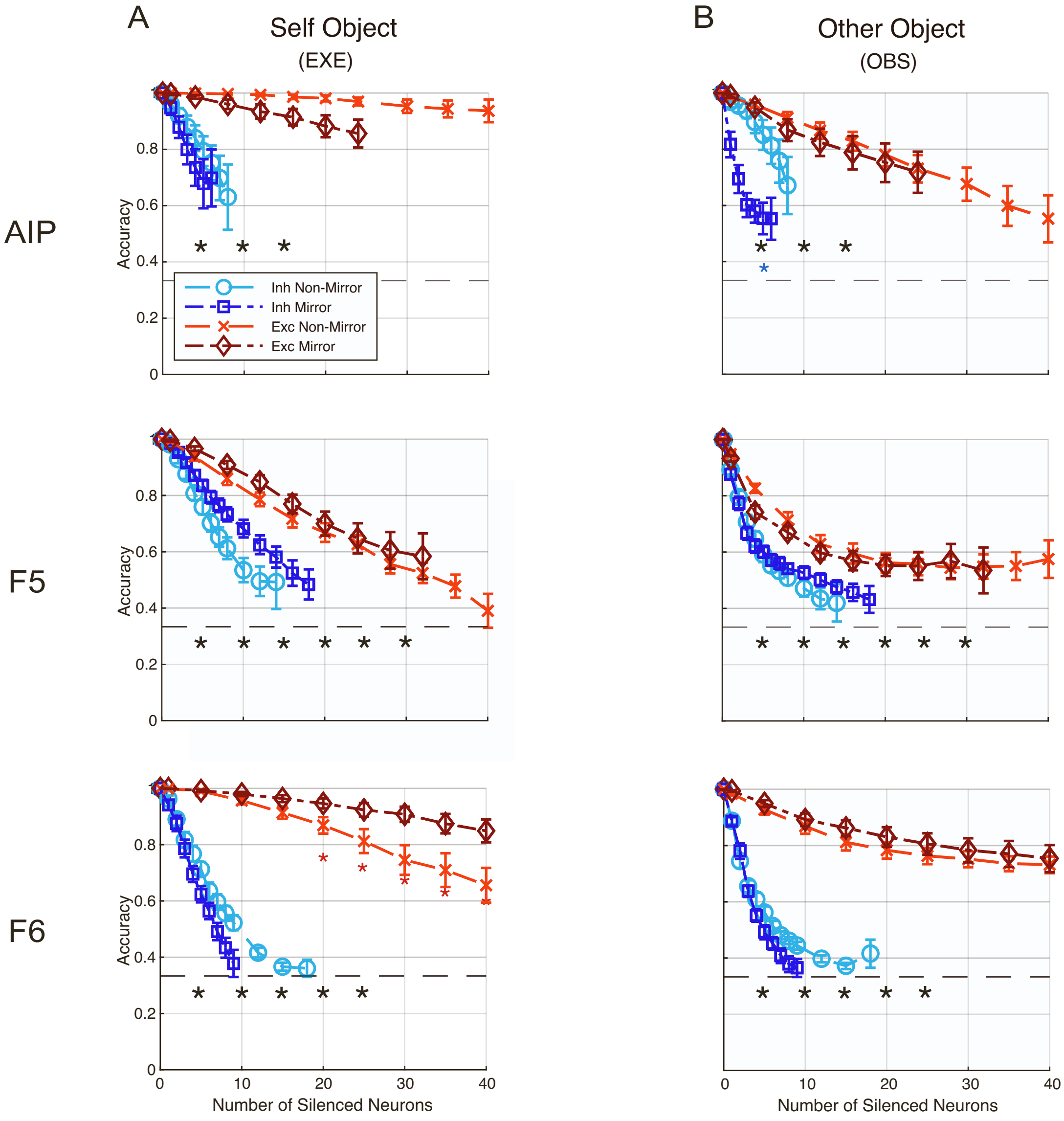

Supplemental Figure 6 | Silencing effects on object decoding during execution and observation, related to Figure 3. (A) Accuracy of self-object and (B) other-object decoding as a function of the number of silenced neurons, grouped by cell type—excitatory (Exc, red) and inhibitory (Inh, blue)—and functional class—mirror (MN, dark shades) and non-mirror (nM, light shades). Data represent means across 20 RNNs and 50 random silencing samples per network; error bars indicate 95% confidence intervals across networks. Black asterisks mark significant differences (permutation test, $p < 0.05$) between inhibitory and excitatory neurons regardless of functional class, red asterisks mark significant differences between excitatory mirror and non-mirror neurons, and blue asterisks mark significant differences between inhibitory mirror and non-mirror neurons.

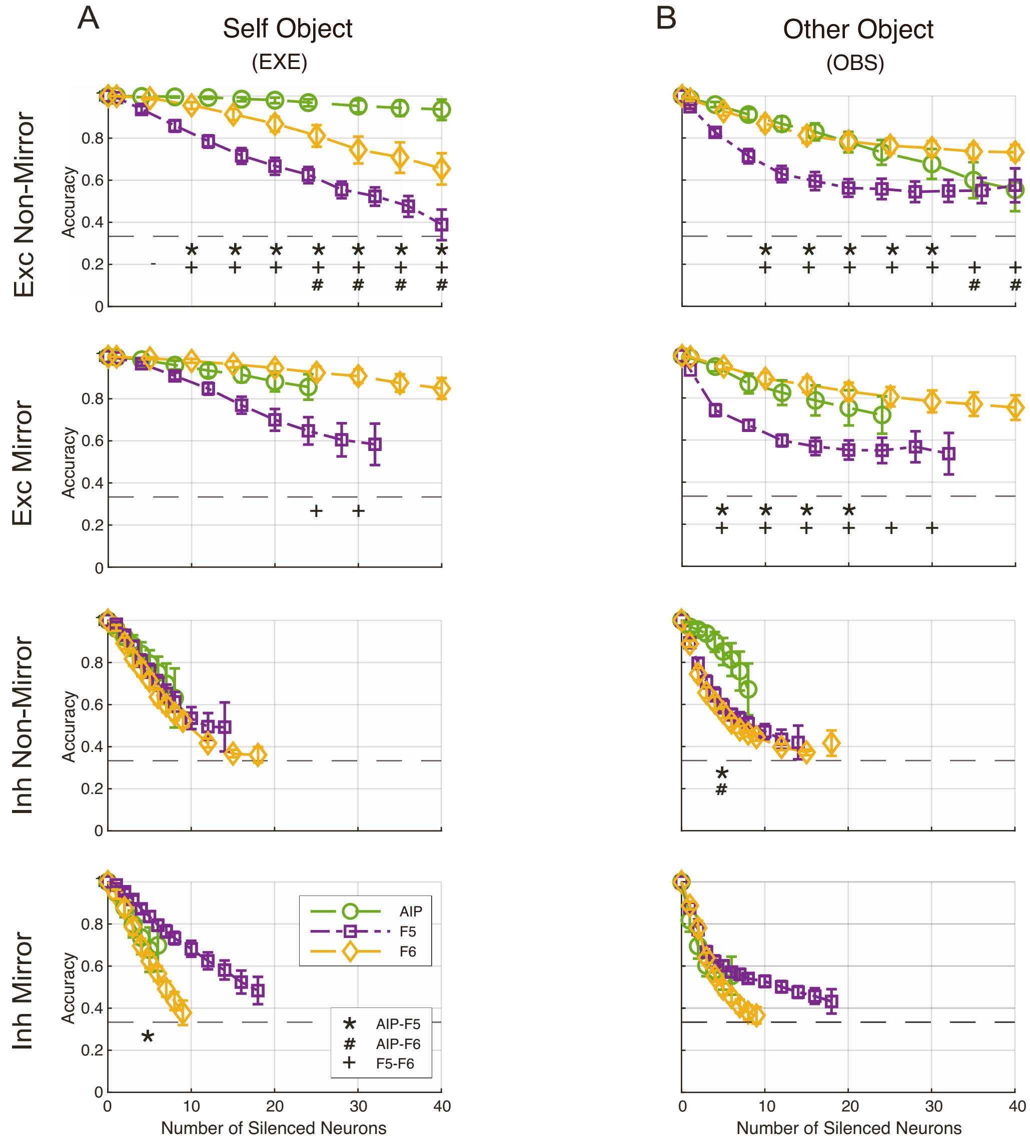

Supplemental Figure 7 | Regional comparison of silencing effects for object decoding across cell classes, related to Figure 3. (A) Classifier accuracy for self-object and (B) other-object decoding as a function of the number of silenced neurons in AIP (green), F5 (violet), and F6 (yellow), divided by cell class: excitatory non-mirror (top), excitatory mirror (upper middle), inhibitory non-mirror (lower middle), and inhibitory mirror (bottom). Data represent means across 20 RNNs and 50 random silencing samples per network; error bars indicate 95% confidence intervals across networks. Asterisks (*) indicate significant differences (permutation test, $p < 0.05$) between AIP and F5, hash symbols (#) between AIP and F6, and plus signs (+) between F5 and F6.

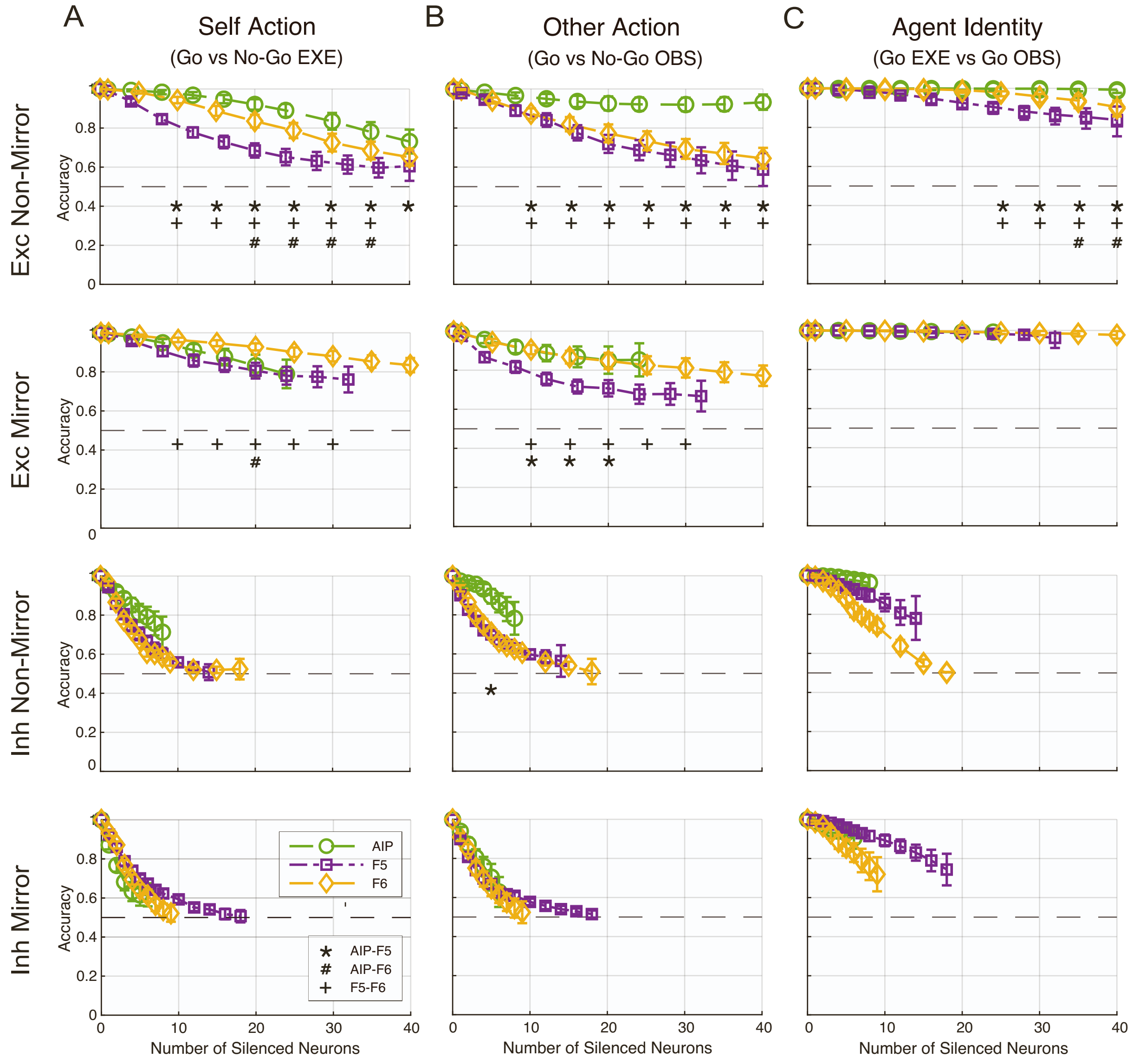

Supplemental Figure 8 | Regional comparison of silencing effects across cell classes, related to Figure 3. (A) Classifier accuracy for self-action, (B) other-action, and (C) agent identity decoding as a function of the number of silenced neurons in AIP (green), F5 (violet), and F6 (yellow), divided by cell class: excitatory non-mirror (top), excitatory mirror (upper middle), inhibitory non-mirror (lower middle), and inhibitory mirror (bottom). Data represent means across 20 RNNs and 50 random silencing samples per network; error bars indicate 95% confidence intervals across networks. Asterisks (*) indicate significant differences (permutation test, $p < 0.05$) between AIP and F5, hash symbols (#) between AIP and F6, and plus signs (+) between F5 and F6.

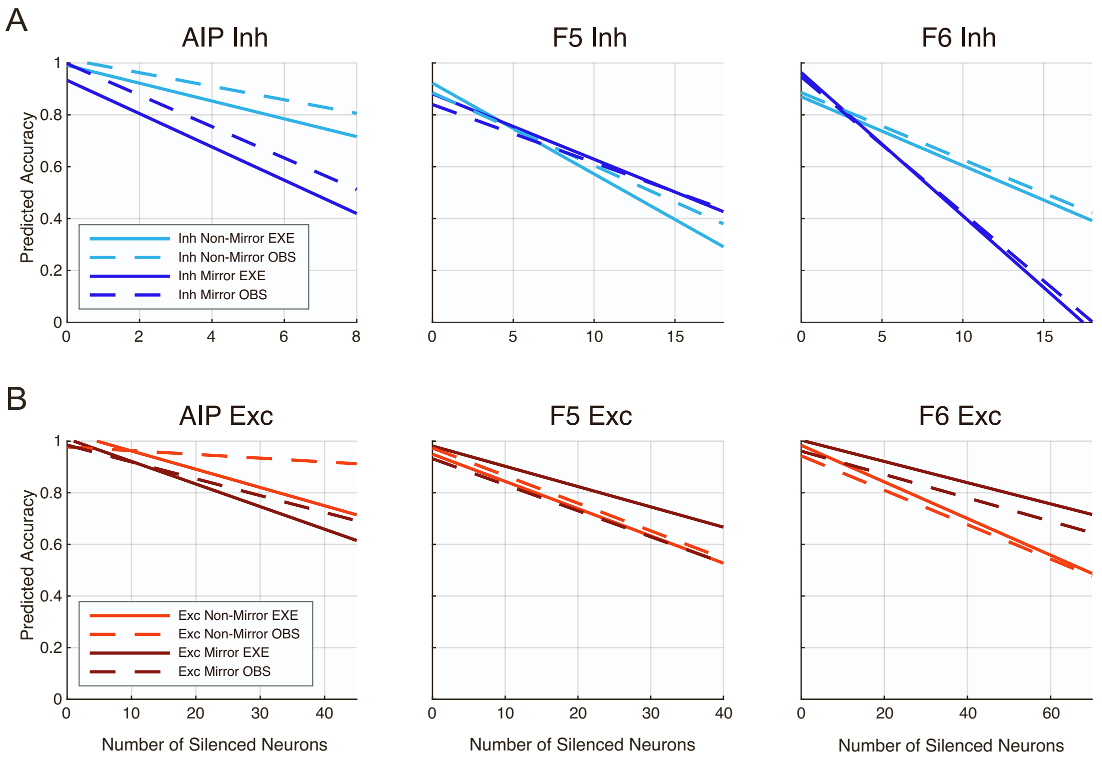

Supplemental Figure 9 | LME-predicted interaction plots for action decoding, related to Figure 3. Fixed-effect predictions from the linear mixed-effects model are shown separately for inhibitory (A) and excitatory (B) populations in AIP (left), F5 (middle), and F6 (right). The x-axis indicates the number of silenced neurons, and the y-axis indicates the decoding accuracy predicted by the model. Curves show model predictions for the four combinations of functional class and action context: non-mirror EXE, non-mirror OBS, mirror EXE, and mirror OBS. EXE refers to self-action decoding and OBS to observed-action decoding in the Go condition. Solid lines denote EXE and dashed lines denote OBS; light and dark shades indicate non-mirror and mirror populations, respectively. These plots visualize whether the mirror/non-mirror difference changes between action contexts and across progressive silencing levels.

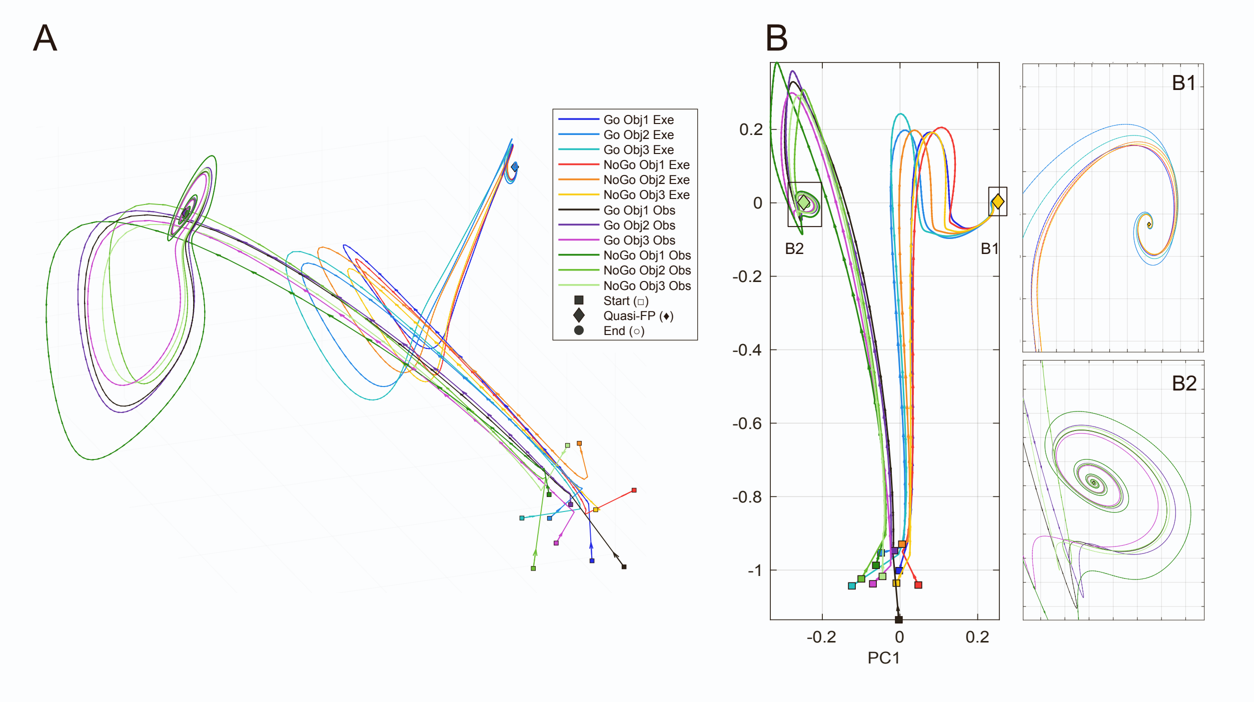

Supplemental Figure 10 | Population trajectories in 3D (A) and 2D (B) PCA space for a representative trained network simulated without external input or noise, related to the execution/observation input structure described in the main text. Each line represents one of the twelve initial conditions (1–6: Execution, 7–12: Observation), generated as small perturbations around the corresponding experimental states. For visual clarity, the initial simulation steps were omitted. Markers indicate the start (□), quasi–fixed point (◇), and end (○) of each trajectory. Trajectories converge toward two compact regions corresponding to the Execution (B1) and Observation (B2) conditions, respectively. This indicates that the separation between execution and observation is not imposed exclusively by the context-specific input weights, but is also retained in the learned recurrent dynamics of the model.

Supplemental Tables

Supplemental Table 1 | Performance metrics for RNN reconstruction, related to Figure 1. Global population-level Pearson correlation coefficient (R) and root mean squared error (RMSE) between experimental and RNN-generated firing rates for each of the 20 independently trained networks. Average values are reported as mean $\pm$ standard deviation across networks.

RNN index	R	RMSE
1	0.9484	0.0184
2	0.9478	0.0186
3	0.9473	0.0187
4	0.9379	0.0202
5	0.9481	0.0185
6	0.9409	0.0198
7	0.9462	0.0187
8	0.9483	0.0185
9	0.9386	0.0200
10	0.9445	0.0191
11	0.9402	0.0198
12	0.9443	0.0191
13	0.9488	0.0185
14	0.9204	0.0232
15	0.9403	0.0203
16	0.9376	0.0205
17	0.9482	0.0187
18	0.9345	0.0213
19	0.9431	0.0195
20	0.9461	0.0189
Mean $\pm$ std	0.943 $\pm$ 0.007	0.0195 $\pm$ 0.0012

Supplemental Table 2 | Inter-area connectivity percentages, related to Figure 2. Values represent the fraction of existing (nonzero) synaptic connections relative to all possible ones between each pair of cortical areas (AIP, F5, F6), excluding projections fixed to zero by design. Data are averaged across 20 independently trained networks (mean ± SD).

Connection	Value (%)	SD (%)
AIP $\rightarrow$ AIP	5.66	0.33
AIP $\rightarrow$ F5	4.26	0.39
AIP $\rightarrow$ F6	2.94	0.34
F5 $\rightarrow$ AIP	0.90	0.12
F5 $\rightarrow$ F5	8.34	0.40
F5 $\rightarrow$ F6	2.46	0.21
F6 $\rightarrow$ AIP	0.94	0.11
F6 $\rightarrow$ F5	4.58	0.30
F6 $\rightarrow$ F6	5.21	0.25

Supplemental Table 3 | Intra-area connectivity percentages, related to Figure 2. Values represent the fraction of existing (nonzero) synaptic connections relative to all possible ones within each cortical area, computed separately by cell type—excitatory (Exc) and inhibitory (Inh)—and functional class—mirror (M) and non-mirror (nM). Data are averaged across 20 independently trained networks (mean ± SD).

Connection	Value (%)	SD (%)
**AIP**		
Inh → Exc	32.34	1.81
Exc → Inh	1.08	0.47
nM → M	4.91	0.51
M → nM	7.60	0.44
M Inh → M Inh	27.22	5.07
nM Inh → nM Inh	22.03	4.62
M Exc → M Exc	0.78	0.37
nM Exc → nM Exc	0.50	0.22
**F5**		
Inh → Exc	14.77	0.79
Exc → Inh	7.22	0.57
nM → M	8.08	0.45
M → nM	8.30	0.58
M Inh → M Inh	18.39	1.69
nM Inh → nM Inh	17.63	1.62
M Exc → M Exc	4.14	0.50
nM Exc → nM Exc	4.19	0.54
**F6**		
Inh → Exc	20.95	1.17
Exc → Inh	4.11	0.60
nM → M	5.28	0.20
M → nM	4.73	0.49
M Inh → M Inh	18.02	3.81
nM Inh → nM Inh	18.69	1.97
M Exc → M Exc	1.81	0.28
nM Exc → nM Exc	1.87	0.21

Supplemental Table 4 | Outgoing inter-area connectivity percentages for excitatory mirror and non-mirror neurons, related to Figure 2. Values represent the fraction of existing (nonzero) outgoing synaptic connections directed to each target area, computed separately for excitatory mirror and non-mirror neurons within each source area. Data are averaged across 20 independently trained networks (mean $\pm$ SD).

Connection	Mirror (%)	Non-Mirror (%)
**AIP**		
To F5	4.3 ± 0.4	4.2 ± 0.5
To F6	2.8 ± 0.4	3.0 ± 0.4
To F5+F6	3.4 ± 0.3	3.5 ± 0.4
**F5**		
To AIP	0.9 ± 0.2	0.9 ± 0.2
To F6	2.3 ± 0.3	2.6 ± 0.2
To AIP+F6	1.8 ± 0.2	2.0 ± 0.2
**F6**		
To AIP	0.9 ± 0.1	1.0 ± 0.1
To F5	4.5 ± 0.3	4.7 ± 0.4
To AIP+F5	2.9 ± 0.2	3.0 ± 0.2

Supplemental Table 5 | Linear mixed-effects marginal interaction analysis for action decoding, related to Figure 9. Models were fitted separately for each area and cell type using the formula: *Accuracy* $\sim$ *NumSilenced*$_c$ $\times$ *Context* $\times$ *Functional Class* $+ (1|$*Network*$)$. Context refers to EXE/self-action and OBS/other-action decoding; functional class refers to non-mirror and mirror neurons. For each model, Δ_{int} denotes the LME-derived marginal interaction contrast $[(M - nM)_{\text{OBS}} - (M - nM)_{\text{EXE}}]$, averaged over the common silencing range; the associated p-value is reported in parentheses. Positive values indicate a larger mirror-versus-non-mirror contrast in OBS than in EXE, whereas negative values indicate a smaller mirror-versus-non-mirror contrast in OBS than in EXE. The last column reports the p-value for the *NumSilenced* $\times$ *Context* $\times$ *Functional Class* interaction, indicating whether the interaction varied along the progressive-silencing curve.

Area	Cell Type	$\Delta_{\text{int}} = [(M - nM)_{\text{OBS}} - (M - nM)_{\text{EXE}}]$	p(NumSil. $\times$ Context $\times$ Funct. Class)
AIP	Inh	$+0.026$ ($p = 0.063$)	0.450
AIP	Exc	-0.010 ($p = 0.376$)	0.002
F5	Inh	-0.033 ($p = 3.64 \times 10^{-4}$)	0.035
F5	Exc	-0.106 ($p = 5.63 \times 10^{-18}$)	0.040
F6	Inh	-0.023 ($p = 0.038$)	0.508
F6	Exc	-0.029 ($p = 0.0017$)	0.046

Supplemental Notes

Signal Reconstruction and Performance Metrics

We constructed a continuous-rate recurrent neural network (RNN) model comprising 355 units, each corresponding one-to-one to a recorded neuron in the macaque AIP, F5, and F6 areas. Each network was trained to reproduce the firing rates of all neurons across the 12 task conditions (3 objects × 2 Go/No-Go × 2 execution/observation), encompassing the full behavioral design of the experiment.

The network dynamics followed the rate equations reported in the main text (Eqs. 1-2), combining task-specific visual and auditory inputs to replicate the experimentally recorded activity. Training minimized the deviation between simulated firing rates r and empirical data $\hat{r}$, aggregated across all neurons, time points, and task conditions (Eq. 6, main text).

Reconstruction metrics. To quantify reconstruction accuracy, we computed the root-mean-squared error (RMSE) between model-generated and experimental firing rates:

$$\mathrm{RMSE} = \sqrt{\frac{1}{12\,T\,N}\sum_{task=1}^{12}\sum_{t=1}^{T}\sum_{i=1}^{N}\left(r_{i,t,task} - \hat{r}_{i,t,task}\right)^2}. \tag{1}$$

The temporal fidelity between the reconstructed and experimental signals was assessed using a global Pearson correlation coefficient.

$$R = \frac{1}{12}\sum_{task=1}^{12}\frac{\sum_{i,t}(r_{i,t,task} - \bar{r}_{task})(\hat{r}_{i,t,task} - \bar{\hat{r}}_{task})}{\sqrt{\sum_{i,t}(r_{i,t,task} - \bar{r}_{task})^2}\sqrt{\sum_{i,t}(\hat{r}_{i,t,task} - \bar{\hat{r}}_{task})^2}}, \tag{2}$$

where $\bar{r}_{task}$ and $\bar{\hat{r}}_{task}$ denote task-wise mean activity across neurons and time points.

We adopted a global Pearson correlation coefficient rather than averaging neuron-wise correlations, as the latter can be strongly biased by neurons with very low firing rates, which were frequent in our dataset. For such neurons, even minimal fluctuations can yield disproportionately low or negative correlations despite qualitatively accurate reconstructions. By pooling all neurons and time points into a single vector per task, the global correlation provides a more stable and representative measure of population-level fidelity between model and experimental activity.

Robustness across network realizations. To ensure robustness, we independently trained 20 networks with distinct random initializations. Evaluation metrics were averaged across these realizations, yielding high-fidelity reconstructions of both single-neuron and population-level activity (mean ± SD: $\mathrm{RMSE} = 0.0195 \pm 0.0012$; $R = 0.943 \pm 0.007$; Supplemental Table 1).

Population-level trajectory structure. Beyond these quantitative measures, we also compared the structure of the neural trajectories through Principal Component Analysis (PCA). As reported in the main text, the first two principal components explained (*mean* $\pm$ SD) $60.4 \pm 4.4\%$ of the variance in the experimental activity and $77.5 \pm 4.7\%$ in the synthetic activity, while the first three explained $70.6 \pm 5.0\%$ and $87.7 \pm 3.2\%$, respectively. These results indicate that the leading components captured a substantial fraction of the population dynamics in both cases, although the synthetic activity was more strongly concentrated along the dominant dimensions. This qualitative agreement is illustrated in Supplemental Figure 1.

Connectivity Analysis and Task-Specific Training

The recurrent structure of each trained network is defined by the directed weight matrix W, whose element w_{ij} denotes the synaptic connection from presynaptic neuron j (column) to postsynaptic neuron i (row). Each unit was assigned as excitatory or inhibitory based on spike-waveform classification of the

corresponding recorded neuron, Dale's principle was enforced (purely excitatory or inhibitory outputs), and inhibitory projections were restricted to targets within the same cortical area (AIP, F5, F6).

The matrix W was initialized as fully connected and evolved through training toward a sparse organization that was highly consistent across networks.

Network-level connectivity measures. For network-level statistics, connectivity matrices were binarized prior to analysis by setting all nonzero weights to 1, without applying any additional threshold. This procedure preserved the full pattern of learned connections without imposing arbitrary cutoffs. Connection densities ("overall connectivity") were computed, for each area pair, as the fraction of existing (nonzero) links relative to all possible links, excluding edges constrained to be absent by design (e.g., self-connections and inter-areal inhibitory projections).

For each of the 20 independently trained networks, within- and between-area connection probabilities were measured for each pathway (e.g., AIP$\rightarrow$F5, within-F5, etc.) and then averaged across networks; detailed values and confidence intervals across networks are reported in Supplemental Tables 2–3. To further assess whether mirror and non-mirror neurons differed in their long-range connectivity profiles, we quantified outgoing inter-area connectivity separately for excitatory mirror and non-mirror populations in AIP, F5, and F6 (Supplemental Table 4). The resulting connection probabilities were broadly similar across the two functional classes, with only modest quantitative differences and no evidence of a clear segregation of inter-area projections.

Node-level graph metrics. Node-level metrics were evaluated on the binarized graphs and included in-degree and out-degree (number of incoming and outgoing connections), local efficiency, and betweenness centrality. Unless otherwise noted, the statistical unit was the single neuron: for each recorded neuron, the corresponding metric was computed in every network instance and then averaged across the 20 networks before group comparisons. Groupwise differences were assessed using the Mann–Whitney U test. Comparisons were organized along three factors: (i) cell type (excitatory vs inhibitory), (ii) functional class (mirror vs non-mirror), and (iii) anatomical area (AIP, F5, F6).

Across networks, inhibitory neurons consistently showed higher in-degree and out-degree than excitatory neurons, consistent with dense local inhibition and a stabilizing role in circuit dynamics. Across cortical areas, F5 exhibited the highest average degree, whereas mirror and non-mirror neurons displayed broadly comparable connectivity profiles across regions (Supplemental Figure 2A–B).

The recurrent architecture was not initialized as an unconstrained random network, but incorporated biologically motivated constraints, including Dale's principle and the restriction of inhibitory projections within each cortical area. We therefore first examined local efficiency and betweenness centrality before training, and then quantified how these metrics changed during optimization using the post-minus-pre difference for each neuron and each independently trained network.

The pre-training analysis showed that inhibitory neurons already displayed higher local efficiency than excitatory neurons, consistent with the imposed local-connectivity constraint. In contrast, inhibitory neurons showed minimal betweenness centrality before training, indicating that their prominent centrality in the trained networks was not directly imposed by the initialization (Supplemental Figure 2C).

The post-minus-pre analysis further showed that training differentially reshaped the topology of excitatory and inhibitory neurons. Local efficiency decreased after training in both cell types, consistent with the sparsification of the initially dense recurrent matrix; however, this reduction was weaker in inhibitory neurons and stronger in excitatory neurons. Betweenness centrality showed the opposite pattern: although it increased after training in both cell types, this increase was markedly stronger in inhibitory than excitatory neurons (Supplemental Figure 2D). Thus, the final inhibitory topology reflected both the imposed local-connectivity constraint and training-dependent reorganization, with inhibitory neurons preserving stronger local neighborhood structure while acquiring a more central role within the recurrent architecture.

Task-specific training controls. To evaluate robustness and context dependence, we additionally trained separate models on execution-only and observation-only datasets. Despite the restricted training sets, these models preserved the main organizational features observed under joint training, including overall synaptic

density and the predominance of intra-areal connectivity. These control analyses support the stability of the learned architecture across behavioral contexts (Supplemental Figure 2E–F).

Supplementary analysis of learned input weights

Because execution and observation were implemented through distinct input matrices acting on a shared recurrent architecture, we performed an additional analysis of the learned input weights to assess how the two contexts differed at the level of external drive to different neuronal populations.

To reduce dimensionality, the ten input channels were grouped into four anatomically defined streams: auditory input to F6, visual input to AIP, auditory input to F5, and visual input to F5. All the analyses described below were performed on these macro-channels, first on signed input weights and then repeated on the absolute values of the same weights.

Differences across neuronal classes. We first tested whether different neuronal classes received systematically different inputs within each context. For each neuron, input weights were averaged across the 20 independently trained networks, and comparisons were performed between mirror and non-mirror neurons, as well as between excitatory and inhibitory neurons, within each area and modality. Statistical comparisons were performed using Mann–Whitney U tests.

Only a small number of significant differences were detected in the signed input weights. In particular, during observation, mirror neurons received stronger visual input than non-mirror neurons in both AIP and F5. We also tested for differences between excitatory and inhibitory neurons and found that, during execution, excitatory neurons in F5 received weaker auditory input than inhibitory neurons. No other significant differences were detected across neuronal classes. When the same analysis was repeated on the absolute values of the input weights, these class-dependent differences were no longer observed, indicating that the few effects detected in the signed weights depended on the signed structure of the inputs rather than on a robust difference in input magnitude.

Context-dependent differences between EXE and OBS. We then directly compared execution- and observation-related input weights within each neuronal population. For each network and each population, mean input weights were computed for the relevant modality and compared between EXE and OBS conditions using paired Wilcoxon signed-rank tests across networks.

These comparisons revealed EXE–OBS differences in mean input weight in all cases except the auditory input for non-mirror neurons in F6 (Supplemental Figure 3A). The clearest effects were observed in visual inputs to AIP and F5. In AIP, the signed weights were close to zero or weakly positive during execution and negative during observation, for both mirror and non-mirror neurons. In F5 visual inputs, EXE–OBS differences were also evident, although with different average magnitudes across mirror and non-mirror populations. At the area level, comparisons across networks showed that the absolute magnitude of visual input was consistently larger in AIP than in F5, whereas the absolute magnitude of auditory input was consistently larger in F6 than in F5, in both execution and observation. Repeating the same within-population comparisons on the absolute values of the input weights yielded the same qualitative pattern, suggesting that the EXE–OBS differences do not depend only on sign changes but also reflect differences in input magnitude (Supplemental Figure 3B).

Cosine similarity of input patterns. To further characterize the relationship between EXE and OBS inputs, we computed the cosine similarity between execution- and observation-related input vectors within each population, separately for visual and auditory channels. For each network, cosine similarity was computed across neurons within the corresponding population, and summary statistics were then obtained across networks.

For signed input weights, cosine similarity values were generally low or intermediate across populations, indicating only partial similarity between EXE and OBS input patterns (Supplemental Figure 3A). Significant differences in cosine similarity between mirror and non-mirror populations were observed for visual channels, where mirror populations in AIP and F5 showed higher similarity values than the corresponding non-mirror

populations. By contrast, cosine similarities for auditory inputs were comparable between mirror and non-mirror populations.

When cosine similarity was recomputed on the absolute values of the input weights, values were markedly higher overall, and no significant differences between mirror and non-mirror populations were observed (Supplemental Figure 3B). This indicates that the mirror/non-mirror differences seen in the signed cosine analysis were driven by the signed structure of the input vectors, rather than by a robust separation in their absolute magnitude.

Overall, these analyses indicate that execution and observation are associated with robust differences in input weights across populations, whereas differences between neuronal classes are limited and depend primarily on the signed structure of the learned inputs.

Functional Contribution of Neuronal Subpopulations via Silencing

To investigate the functional role of specific neuronal subpopulations, we implemented an in silico silencing procedure in which the firing rates of selected neurons were clamped to zero during network activity generation. This approach provides a controlled perturbation of identified neuronal groups at cell-type resolution, conceptually analogous to selective optogenetic or pharmacological perturbations, while allowing a finer level of specificity than is currently achievable in vivo.

Decoding framework and silencing procedure. To assess how different subpopulations contribute to task-relevant information, we trained a bidirectional Long Short-Term Memory (LSTM) classifier on intact network activity to capture several task-relevant distinctions: "self action" and "other action" decoding, distinguishing Go and No-Go trials during execution and observation, respectively; "self object" and "other object" decoding, discriminating the three objects in Go trials separately for the two contexts; and "agent identity" decoding, differentiating between performed versus observed Go trials. Each classifier was trained on a fixed 0.8-s window following the offset of the Go/No-Go cue, corresponding to the epoch of strongest task-related modulation observed experimentally.

The silencing procedure was systematically applied across all networks by randomly selecting 50 neuron subsets from each target subpopulation for all tested conditions. Classifier accuracy was assessed for each subset, with final results obtained by averaging across both the silencing samples and the 20 network realizations. To characterize silencing effects, we progressively silenced increasing numbers of neurons within each defined subpopulation, examining all combinations of excitatory/inhibitory cell types and mirror/non-mirror properties, with separate analyses conducted for each cortical area (AIP, F5, F6). This approach allowed us to track the resulting changes in classifier accuracy while maintaining anatomical specificity. Here we report the effects on self-object and other-object decoding (Supplemental Figure 6A–B). To reveal area-specific contributions, we also organized the results to compare how silencing each defined subpopulation—excitatory non-mirror, excitatory mirror, inhibitory non-mirror, and inhibitory mirror—in the three cortical areas impacted decoding performance (Supplemental Figures 7–8).

Absolute and percentage-based silencing analyses. Given the substantial variability in subpopulation sizes, ranging from just 6 inhibitory mirror neurons in AIP to over 70 excitatory non-mirror neurons in F6, we additionally analyzed silencing effects using percentage-based normalization (Supplemental Figures 4–5). This approach complements the absolute-number silencing analysis by enabling more comparable assessments across populations with markedly different sizes. The qualitative agreement between absolute and percentage-based silencing results supports the robustness of the main effects across analysis formats.

Statistical analysis. Significance of silencing effects was assessed with pairwise permutation tests (100 iterations) for each comparison and decoding variable. For a given comparison (e.g., inhibitory vs excitatory), we computed the observed difference in accuracy, averaged across the 20 networks and 50 draws. Subpopulation labels were then randomly permuted 100 times; at each iteration, accuracies were recomputed and averaged across networks to form a pooled null distribution of group-level differences. The empirical p-value was defined as the fraction of permutations with a difference $\geq$ the observed effect, with significance

set at $p \leq 0.05$. Ninety-five-percent confidence intervals across networks are also reported to quantify across-model reliability.

We tested for differences between mirror and non-mirror neurons separately within excitatory and inhibitory populations. Additionally, we compared the effects of silencing inhibitory versus excitatory neurons, irrespective of functional class. Finally, each of the four subpopulations—excitatory mirror, inhibitory mirror, excitatory non-mirror, and inhibitory non-mirror—was independently analyzed to assess the impact of silencing across cortical areas. In the figures, black asterisks indicate significant differences between inhibitory and excitatory neurons (regardless of functional class), while red and blue asterisks denote significant differences between mirror and non-mirror neurons within the excitatory and inhibitory populations, respectively. Area-wise comparisons are marked as follows: asterisks (*) indicate significant differences between AIP and F5, hash symbols (#) between AIP and F6, and plus signs (+) between F5 and F6.

To ensure sufficient statistical power and interpretability, comparisons between excitatory and inhibitory populations, regardless of mirror properties, as well as between mirror and non-mirror neurons within the same cell type, were performed when at least 5 neurons per group could be silenced. In these cases, where permitted by subpopulation sizes, silencing levels typically increased in steps of 5 neurons (e.g., 5, 10, 15, ...). For percentage-based analyses, statistical tests were applied starting from a silencing level of at least 35% of the corresponding subpopulation, with increments of approximately 15% at each step.

Permutation testing in percentage-based silencing requires particular caution. When comparing subpopulations of markedly different sizes, silencing the same percentage can correspond to very different absolute numbers of neurons being silenced. While this asymmetry introduces additional sampling variability, the comparison between absolute and percentage-based analyses helps distinguish effects that are robust across silencing schemes from those that may depend more strongly on population size.

Linear mixed-effects analysis of context-dependent mirror/non-mirror differences. Because the point-by-point permutation tests quantify differences between predefined silencing curves at specific perturbation levels within a given decoder/context, they do not directly test whether mirror/non-mirror differences vary across action contexts. We therefore performed a complementary linear mixed-effects (LME) analysis specifically aimed at assessing context-dependent mirror/non-mirror differences in action decoding accuracy.

Models were fitted separately for each cortical area (AIP, F5, F6) and cell type (inhibitory, excitatory). In each model, decoding accuracy was explained as a function of the centered number of silenced neurons, action context, functional class, and their interaction terms, with network identity included as a random intercept:

$$\textit{Accuracy} \sim \textit{NumSilenced}_c \times \textit{Context} \times \textit{Functional Class} + (1|\textit{Network}).$$

Here, Context refers to EXE/self-action and OBS/other-action decoding, whereas Functional Class refers to non-mirror and mirror neurons.

This model was used to assess whether the mirror/non-mirror difference in action decoding changed between EXE and OBS contexts, and whether this context dependence varied along the progressive-silencing curve. To visualize these effects, we plotted fixed-effect LME predictions for each combination of context and functional class across silencing levels (Supplemental Figure 9). These plots show the model-predicted decoding accuracy for non-mirror EXE, non-mirror OBS, mirror EXE, and mirror OBS conditions.

Because *NumSilenced* was modeled as a continuous covariate, the context dependence of mirror/non-mirror differences was summarized across the progressive-silencing curve. For each fitted model, we computed the LME-derived marginal interaction contrast:

$$\Delta_{\text{int}} = [(M - nM)_{\text{OBS}} - (M - nM)_{\text{EXE}}],$$

averaged over the common silencing range from fixed-effect model predictions. This contrast quantifies the average OBS-minus-EXE difference in the mirror/non-mirror gap across the silencing curve. Positive values indicate a larger mirror-versus-non-mirror contrast in OBS than in EXE, whereas negative values indicate a smaller mirror-versus-non-mirror contrast in OBS than in EXE. The corresponding p-value was obtained by testing this LME-derived contrast against zero as a linear combination of the fixed-effect estimates. We also report the p-value for the *NumSilenced* $\times$ *Context* $\times$ *Functional Class* interaction obtained from the fixed-effect tests of the fitted LME, which indicates whether the context-dependent mirror/non-mirror relationship

varied along the progressive-silencing curve.

Overall, the LME-derived marginal interaction analysis indicated that context-dependent mirror/non-mirror differences were not uniform across populations. The marginal interaction contrast was strongest in F5 excitatory neurons ($\Delta_{\rm int} = -0.106$, $p = 5.63 \times 10^{-18}$), indicating that the mirror/non-mirror gap differed markedly between EXE and OBS in this population. Smaller marginal interaction contrasts were also observed in F5 inhibitory neurons ($\Delta_{\rm int} = -0.033$, $p = 3.64 \times 10^{-4}$) and in F6 excitatory neurons ($\Delta_{\rm int} = -0.029$, $p = 0.0017$). In contrast, AIP did not show a significant average marginal interaction contrast in either inhibitory ($\Delta_{\rm int} = 0.026$, $p = 0.063$) or excitatory neurons ($\Delta_{\rm int} = -0.010$, $p = 0.376$).

The three-way interaction with the number of silenced neurons was significant for AIP excitatory ($p = 0.0020$), F5 inhibitory ($p = 0.035$), F5 excitatory ($p = 0.040$), and F6 excitatory populations ($p = 0.046$), indicating that in these cases the context-dependent mirror/non-mirror relationship varied along the progressive-silencing curve. Together, these results show that context-dependent mirror/non-mirror differences in action decoding were population-specific, with the clearest average marginal interaction observed in F5 excitatory neurons.

Context-dependent recurrent dynamics in the absence of external input

To further assess whether the separation between execution and observation was entirely dependent on the use of distinct input weight matrices, we analyzed the dynamics of trained networks after removing both external input and noise. Starting from the twelve initial conditions associated with the experimental task conditions, network activity was projected into PCA space.

As shown in Supplemental Figure 10, trajectories converged toward two compact quasi-stable regions associated with execution- and observation-related initial conditions, respectively. This suggests that the separation between execution and observation is not solely imposed by the context-specific input weights, but is also reflected in the learned recurrent dynamics of the model.
